# Formulation Optimization of *Cladophora glomerata* (Kai)-Based 3D Food Inks Using Xanthan Gum and Konjac Gum for Dysphagia-Friendly Applications

**DOI:** 10.3390/foods15203595

**Published:** 2026-10-10

**Authors:** Yonn Pwint Phyu, Saroat Rawdkuen, Nattaya Konsue

**Affiliations:** 1 Food Science and Technology Program, School of Agro-Industry, Mae Fah Luang University, Chiang Rai 57100, Thailand; 6971401003@lamduan.mfu.ac.th (Y.P.P.); saroat@mfu.ac.th (S.R.); 2 Research Centre of Innovative Food Packing and Biomaterials, Mae Fah Luang University, Chiang Rai 57100, Thailand

**Keywords:** 3D food printing, dysphagia, response surface methodology, xanthan gum, konjac gum, *Cladophora glomerata*, texture-modified foods

## Abstract

Three-dimensional (3D) food printing has emerged as a promising approach for producing texture-modified foods for individuals with dysphagia. However, developing stable food inks from freshwater macroalgae remains challenging due to the need for suitable rheological, structural, and textural properties to ensure printability and swallowing safety. The current study optimized the formulation of *Cladophora glomerata* (Kai)-based 3D food inks using xanthan gum (XG) and konjac gum (KG) for dysphagia-friendly applications. A response surface methodology (RSM) optimal design with 16 formulations evaluated the effects of XG and KG on hardness, adhesiveness, cohesiveness, print accuracy, deformation, and syneresis. Formulations were characterized for rheology, microstructure, and dysphagia suitability according to Japanese texture criteria, and UDF and IDDSI frameworks. The RSM models adequately described hydrocolloid-response relationships (*R*^2^ = 0.8456–0.9750). Selected formulation containing ≥6% hydrocolloid, particularly XG:KG ratios between 1:2 to 3:1 demonstrated superior print accuracy (>94%) and met all three dysphagia classifications. Numerical optimization predicted an optimum formulation of 5.263% XG and 3.188% KG with an overall desirability of 0.91. These findings provide a formulation strategy for developing sustainable, dysphagia-friendly 3D-printed foods from *C. glomerata*.

## 1. Introduction

Dysphagia, defined as difficulty in swallowing, is a significant health concern affecting millions of individuals worldwide. The condition can arise from various etiologies such as neurological disorders (stroke, Parkinson’s disease, dementia), head and neck cancers, structural abnormalities, and age-related physiological decline. Dysphagia is broadly estimated to affect 8% of the general population, representing approximately 590 million people globally [1,2,3]. A systematic review and meta-analysis reported a combined prevalence of 60% across different baseline conditions, though this varies considerably depending on the underlying disease or risk factor [4]. The prevalence is particularly high in hospitalized patients, with rates of 36.5% in acute care settings and up to 42% in acute stroke patients [5,6,7]. Older adults are especially susceptible, with 27% to 91% of institutionalized elderly populations affected. The condition is associated with serious health consequences including malnutrition, dehydration, sarcopenia, aspiration pneumonia, and significantly decreased quality of life [8,9].

To standardize dysphagia-friendly foods, the International Dysphagia Diet Standardization Initiative (IDDSI) developed a globally recognized framework that classifies foods and beverages into eight levels (0–7) according to their textural and rheological properties [1]. Among these, Level 4 (Pureed) foods are characterized by a smooth, cohesive texture that requires no chewing and can be easily deformed under minimal pressure, making them suitable for individuals with moderate-to-severe dysphagia [1,10]. Similarly, Japan established official criteria through the Ministry of Health, Labor and Welfare (MHLW) in 2009, now administered by the Consumer Affairs Agency (CAA), which defines acceptable texture profile analysis (TPA) parameters, including hardness, adhesiveness, and cohesiveness, for dysphagia foods. According to the severity of swallowing impairment, foods are classified into three categories (Regulations I–III), each with specified ranges for these textural properties [11]. In addition, the Universal Design Food (UDF) framework classifies semi-solid foods into four stages based primarily on hardness values determined by TPA [8]. These classification systems serve as essential benchmarks for developing safe and nutritious dysphagia-oriented foods.

Despite the availability of these guidelines, conventional texture-modified foods, particularly pureed diets, remain unappealing in appearance, texture, and taste, often leading to reduced intake and exacerbating malnutrition in dysphagic patients. Three-dimensional (3D) food printing offers a transformative solution by enabling personalized foods with precisely controlled structure, texture, and nutrition. Extrusion-based 3D printing deposits semisolid food inks layer-by-layer, allowing customization of shape, infill density, and texture, and has attracted interest in dysphagia management by transforming unpalatable pureed foods into visually appealing, texture-tailored forms that are both safe to swallow and visually appetizing while enhancing mealtime experience and nutritional quality [12,13]. However, successful 3D food printing requires food inks with specific rheological properties: shear-thinning behavior for smooth extrusion, sufficient yield stress for shape retention, and rapid viscosity recovery after deposition [14,15].

Hydrocolloids play a crucial role in modifying the rheological and structural properties of 3D food inks [16]. Among these, xanthan gum (XG) and konjac gum (KG) are particularly promising due to their synergistic interactions thickening and gelling properties, and established safety [17,18,19,20,21]. XG exhibits a rigid helical structure that provides viscosity and shear-thinning behavior, while KG, a glucomannan, offers flexibility and gel-forming capabilities [19,22]. Together, they form synergistic networks via hydrogen bonding and van der Waals interactions, which enhance viscosity, shear-thinning, and post-extrusion recovery. XG-KG blends improve printability and stability in diverse food systems [13,23] with optimal ratios maximizing gel strength and water retention [20,21,24].

Algae have gained considerable attention as sustainable and nutritious food sources [25,26]. Among freshwater algae, *Cladophora glomerata* Kützing locally known as “Kai” or river weed in Northern Thailand has been traditionally consumed and recognized for its health benefits, including digestive support, appetite stimulation and relief of constipation [27,28]. It is also rich in proteins, essential amino acids, minerals (Ca, K, Mg, Fe), and bioactive compounds with antioxidant, anti-inflammatory, and anti-ulcer activities [29,30,31]. In Thailand, Kai is naturally abundant in the upper Nan watershed and Mekong River from November to March, when water clarity, flow rate, and temperature are optimal [32]. However fresh Kai contains high moisture content (≈90%) and deteriorates rapidly, necessitating preservation through drying. Drying this highly perishable biomass into a shelf-stable powder directly overcomes the seasonal harvesting window and facilitates the availability of raw material beyond the harvesting season. In our previous study, tray drying at 50 °C produced Kai powder with the most favorable balance of nutritional quality, bioactive retention, and techno-functional properties for 3D food printing applications, along with practical scalability [33]. Despite these promising characteristics, dried Kai powder alone does not possess sufficient viscoelastic properties to form stable 3D-printed structures. Formulation modification using hydrocolloids is essential to improve printability, structural stability, and post-extrusion integrity [34].

Although XG–KG blends have been investigated in various food systems, their application and formulation optimization in Kai-based 3D food inks for dysphagia-oriented applications have not been previously reported. Accordingly, this study aimed to: (1) investigate the effects of XG and KG concentrations on the rheological behavior, texture profile, printing performance, and post-printing stability of Kai-based 3D food inks using Response Surface Methodology; (2) determine the predicted optimal formulation that simultaneously satisfies textural, printability, and stability criteria; and (3) assess the suitability of the 3D-printed formulations according to IDDSI Level 4, Japanese texture criteria, and UDF Stage 4 criteria. The findings are expected to provide a scientific basis for developing sustainable, nutritious, and personalized dysphagia-friendly foods using freshwater macroalgal biomass.

## 2. Materials and Methods

### 2.1. Materials

Tray-dried Kai powder, prepared according to the method described in our previous study [33], was used as the base ingredient. Briefly, fresh Kai algae were collected from the Nan River (Nan Province, Thailand) and transported to the laboratory within 24 h under refrigerated conditions (5 °C). The samples were washed, cut into approximately 10 cm pieces, dried at 50 °C for 6 h using a hot-air tray dryer (Kluay Nam Thai Towop, Bangkok, Thailand), ground using a blender, and sieved through a 100-mesh sieve (150 µm) to obtain uniform particle size. The resulting powder was vacuum-packaged and stored at −18 °C until further use. Food-grade XG and KG were obtained from Union Science Co., Ltd. (Chiang Mai, Thailand). Deionized water was used for the preparation of all food ink formulations.

### 2.2. Formulation of Food Ink

Tray-dried Kai powder was blended with XG and KG according to the experimental design (Table 1) at total hydrocolloid concentrations ranging from 3% to 9% (*w*/*w* of Kai powder). Deionized water was then added at a ratio of 7:1 (water: total dry solids, *w*/*w*). The mixture was manually homogenized for 3 min using a spatula to achieve a uniform paste-like consistency while minimizing foam and air entrapment. The prepared food ink was covered with plastic wrap and allowed to equilibrate at room temperature (25 ± 2 °C) for 20 min prior to 3D printing and characterization.

### 2.3. Experimental Design

Before applying RSM, preliminary formulation screening was conducted using a syringe to assess the extrusion behavior of the Kai ink. XG and KG were evaluated individually and in combination at a 1:1 ratio. Based on these observations, a total gum concentration of 3–9% was selected as a practical experimental range for the RSM study. Design-Expert software, version 13 (Stat-Ease, Inc., Minneapolis, MN, USA), was used to develop an optimal custom experimental design, which created dysphagia friendly optimum food ink with maximal printability and minimal syneresis and deformation. XG and KG were set as input factors, ranging from 0 to 9%, with constraints applied to ensure that their total concentration remained between 3% and 9%. A total of 16 formulation combinations were generated and evaluated using the software’s optimization algorithm, as shown in Table 1. TPA (hardness, adhesiveness, and cohesiveness), printability (print accuracy and deformation), and syneresis were used as responses for the optimization of the Kai food ink formulation. The mean values of the measured responses were used as the response values for RSM analysis. Analysis of variance (ANOVA) and model statistics, including the model *F*-value, *p*-value, coefficient of determination (*R*^2^), adjusted *R*^2^, predicted *R*^2^, adequate precision, and lack of fit, were obtained from Design-Expert software. Model selection was primarily based on *R*^2^, adjusted *R*^2^, predicted *R*^2^, and Adequate Precision, with ANOVA used to assess the statistical significance of the fitted models at *p* < 0.05. Model reduction was performed using forward or backward selection, as appropriate for each response, while maintaining model hierarchy. The final selected models were used to describe the relationships between the formulation variables and responses and for subsequent numerical optimization using the desirability function.

### 2.4. Optimization Criteria and Desirability Function

To determine the optimal formulation of XG and KG for 3D printable Kai-based food inks, a multi-response optimization approach was employed using the desirability function method (Table 2). The independent variables (XG and KG concentrations) were constrained within the experimental design range (0–9%, *w*/*w*). The optimization criteria were defined based on the functional requirements for extrusion-based 3D printing and dysphagia-oriented food applications. The target ranges for hardness, adhesiveness, and cohesiveness were selected according to Regulation I (serious swallowing difficulty) of the Japanese dysphagia food criteria, as shown in Table 2. Hardness, adhesiveness, and cohesiveness were set within these target ranges to ensure appropriate textural properties, while print accuracy was maximized to achieve high shape fidelity. Syneresis and deformation were minimized to enhance structural stability and water retention. Each response was assigned an equal level of importance to achieve a balanced optimization outcome. The overall desirability function was then used to identify the optimal combination of XG and KG that simultaneously satisfied all response criteria.

### 2.5. 3D Printing Setup and Fabrication Parameters

#### 2.5.1. 3D Printing Equipment and Conditions

Samples were fabricated using an extrusion-based food 3D printer (Foodini^®^, Natural Machines, Barcelona, Spain). A 1.5 mm nozzle was used for all printing experiments. The line thickness and layer height were both set at 1.4 mm. Printing was performed at a printing speed of 3500 mm/min with an ingredient flow speed of 1.7. The fill factor was set to 1%, and the turning speed factor was maintained at 0.

To improve first-layer deposition, the first ingredient flow was set to 6, with a first ingredient hold of 4.2 mm. The first-layer nozzle height was fixed at 1.4 mm, and the first-layer speed was maintained at 100%. During non-printing movements, a jump height of 3 mm was applied. The ingredient hold and minimum hold distance were set to 3 mm and 10 mm, respectively, while the resume ingredient flow was set to 0. All printing experiments were conducted at room temperature (25 ± 2 °C) without any pre-heating or active thermal control.

#### 2.5.2. Printing Geometry

The printing geometry was designed using Foodini Creator software (Version 4.0.3) integrated with the Foodini^®^ printer. A cuboidal geometry with dimensions of 30 × 40 × 10 mm (width × length × height) was created for the printed samples, corresponding to 7 printed layers. This geometry was selected to facilitate measurement of length, width, and height and to assess dimensional accuracy and retention of the intended shape.

#### 2.5.3. Toolpath Generation

**Figure 1:**
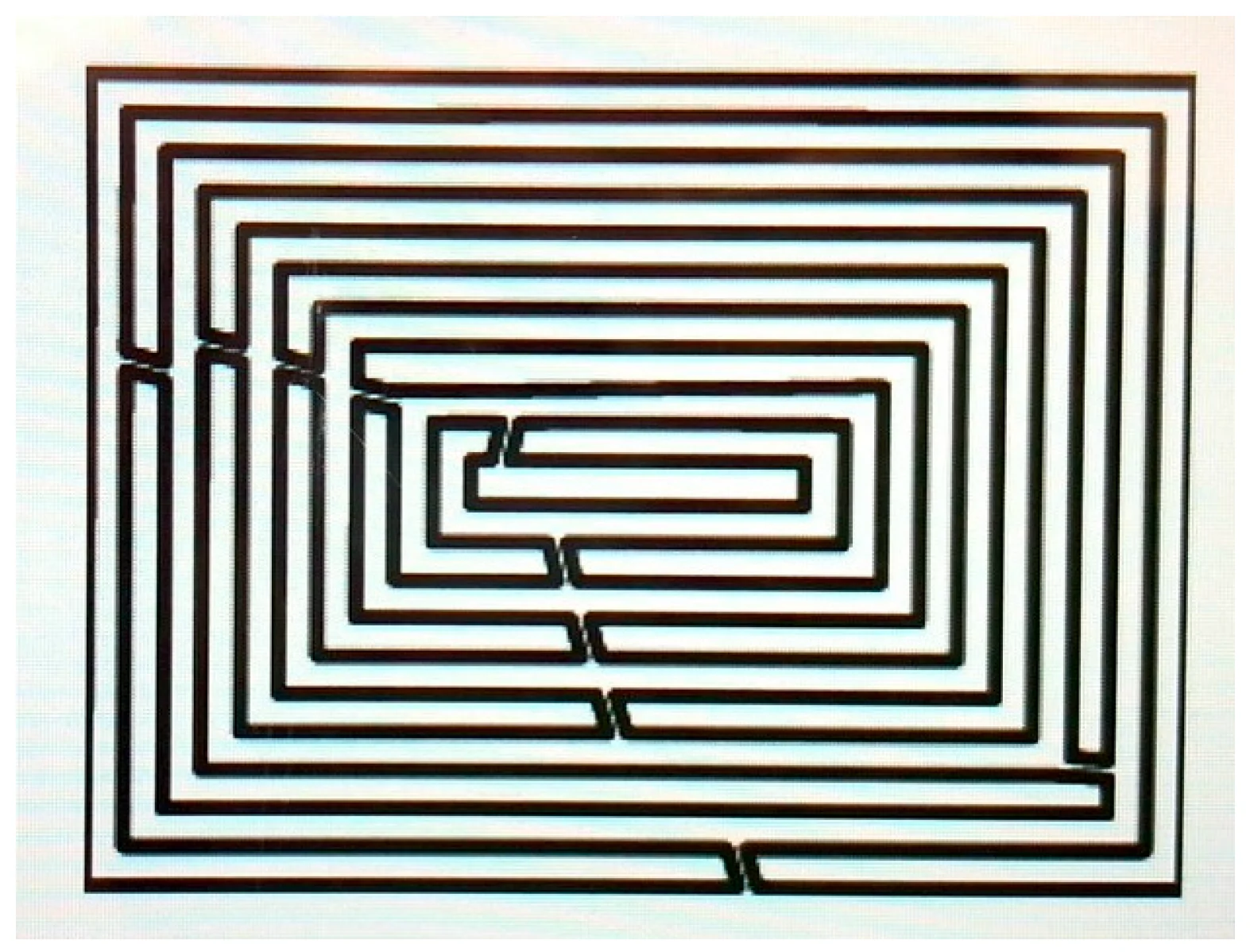
Printing pattern: rectilinear spiral-like deposition pattern.

The toolpath was automatically generated by the Foodini^®^ software using default settings, without manual modification of the deposition strategy. The resulting toolpath produced a continuous rectilinear spiral-like deposition pattern, allowing uniform material extrusion with minimal start–stop events which can be seen in Figure 1. Concentric infill was not applied, and advanced functions including smart fill, outlining, inversion, and stylization were disabled. Smoothing was enabled to improve surface uniformity of the printed samples.

### 2.6. Physical and Mechanical Performance Responses

#### 2.6.1. Print Accuracy and Stability

Printing performance was evaluated in terms of printing accuracy and stability. For printing accuracy, the length, width, and height of the printed samples were measured three times using a vernier caliper and the mean value of corresponding dimensions were used to calculate printing accuracy according to a previous report with slight modification [35]. The printing accuracy was calculated by equation:

(1) $$\mathrm{Print Accuracy}(\%)=\frac{\left(1-\frac{\left|H_{e}-H_{s}\right|}{H_{s}}\right)+\left(1-\frac{\left|L_{e}-L_{s}\right|}{L_{s}}\right)+\left(1-\frac{\left|W_{e}-W_{s}\right|}{W_{s}}\right)+\left(1-\frac{\left|P_{e}-P_{s}\right|}{P_{s}}\right)}{4}\times 100$$

where *H_e_* represents the height of the 3D-printed product and *H_s_* is the height of the reference model. *L_e_* and *L_s_* denote the length of the printed product and model, respectively, while *W_e_* and *W_s_* refer to the width of the printed product and model. In addition, *P_e_* indicates the number of complete layers in the printed product, whereas *P_s_* corresponds to the number of layers in the model. The relative deviation structure in Equation 1 cancels out all physical units, converting the measurements into unitless ratios between 0.0 and 1.0 to ensure a balanced average.

Printing stability was evaluated by measuring the length, width, and height of the printed cuboids using vernier calipers. Each dimension was measured 3 times before storage and remeasured at the same point after storage at 4 °C for 24 h. The mean value of dimensions were used to calculate the deformation rate using Equation (2).

(2) $$\mathrm{Deformation rate}\left(\%\right)=\frac{\left|H_{b}-H_{a}\right|}{H_{b}}\times 100$$

#### 2.6.2. Syneresis of Food Inks

**Figure 2:**
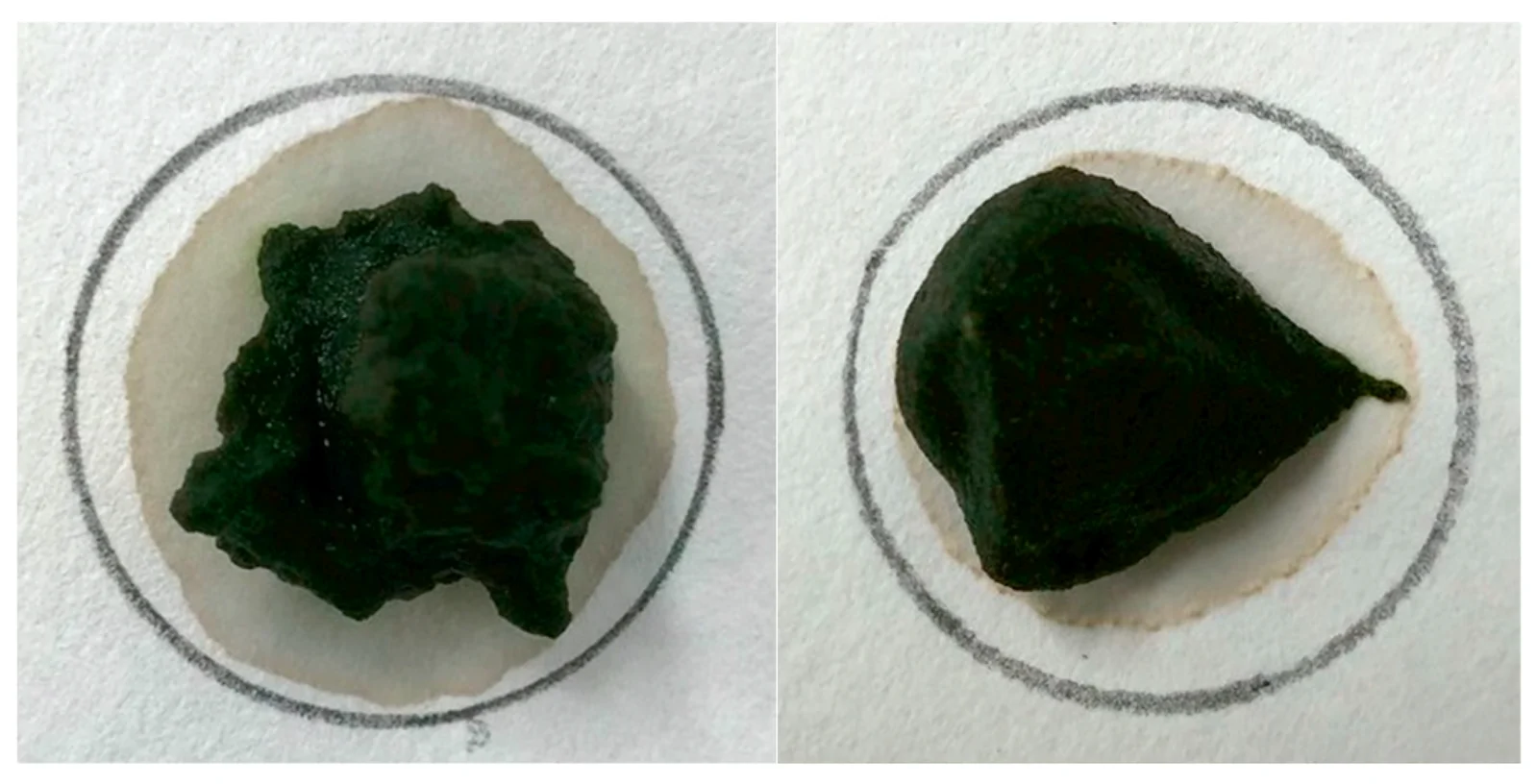
Syneresis measurement using the filter-paper method.

Syneresis was studied using the filter-paper blotting method that previously reported by Zhang et al. (2022) [36]. The experiment was conducted by placing 1 g of protein ink at the center of a Whatman filter paper No. 4 within a 1 cm radius circle (Figure 2). The food inks were left undisturbed for 30 min to allow liquid spreading, after which they were photographed and the diameter of the liquid spread zone was measured. The diameter was measured in triplicate, and the mean diameter was used to calculate the spread area, assuming a circular spread pattern. The measurements were performed in triplicate for each experimental run, and the results were expressed as mean ± SD.

#### 2.6.3. Texture Properties

Texture was analyzed using texture analyzer (TEX-100N; Japan Instrumentation System Co., Ltd., Nara, Japan). Texture profile analysis was selected as testing method. Cell load was set at 100 N and the cylindrical probe with a diameter of approximately 13 mm was used. The samples were pressed twice with 50% strain. The move speed and test speed of probe were set to 600 and 120 mm/min, respectively. Hardness, adhesiveness and cohesiveness were calculated using TEX-PCI software. The calculation setting was set at 5% fragility and 314.15 mm^2^ of Jig area. The measurements were performed in triplicate, and the results were expressed as mean ± SD.

### 2.7. Dysphagia Safety Suitability Assessment

#### 2.7.1. IDDSI Test

To assign each sample to an IDDSI category, two qualitative tests were performed following the IDDSI guidelines. For the fork drip test, a portion of the ink sample was lifted with a standard fork, and the extent to which the material passed through the prongs was observed. This flow behavior was then matched against the descriptions provided for each food level. The spoon tilt test was carried out by taking a teaspoonful of the sample, holding the spoon motionless over a plate, then slowly inclining it to one side and delivering a gentle flick. The response of the sample during this procedure was compared with the IDDSI criteria. During this procedure, the behavior of the ink sample was monitored and compared with IDDSI descriptions [13].

#### 2.7.2. Japanese Texture Criteria and UDF Classification

TPA results, including hardness, adhesiveness, and cohesiveness, were compared with the Japanese texture criteria for foods intended for individuals with swallowing difficulty (Ministry of Health, Labour and Welfare, Government of Japan, 2009; Consumer Affairs Agency, Government of Japan, 2010) [11]. In addition, hardness values were compared with the UDF classification criteria to determine the corresponding dysphagia food stage [8,37].

### 2.8. Rheology Characterization

A HAAKE MARS III rheometer (Thermo Scientific, Bremen, Germany) fitted with a 35 mm parallel plate system was used to characterize the rheological behavior of the samples. The Kai algae ink (1 g) was loaded onto the lower plate, and the parallel plate gap was set to 1 mm. To prevent moisture loss and maintain a stable temperature, a solvent trap was used during testing. Prior to data acquisition, each sample was held at 25 °C for 5 min to achieve thermal equilibrium. Steady-state flow curves were recorded across a shear rate sweep from 0.1 to 100 s^−1^. Yield behavior was evaluated using an oscillatory stress sweep at a fixed frequency of 1 Hz, with the applied shear stress increased from 1 to 2000 Pa [13]. The crossover point between the storage modulus (*G*′) and loss modulus (*G*″) was taken as the operational yield stress. The steady shear data were fitted to the Power Law (Ostwald–de Waele) model [38] to describe the flow characteristics:

(3) $$\eta =K{\overset{\cdot }{\gamma }}^{n-1}$$

where *η* is the apparent viscosity (Pa·s), *K* is the consistency index (Pa·s^n^), $\overset{\cdot }{\gamma }$ is the shear rate (s^−1^) and *n* is the flow behavior index (dimensionless).

### 2.9. Determination of Microstructure (SEM)

Microstructural analysis was performed using a scanning electron microscope. Freeze-dried printed Kai samples were cut into small cubes (less than 1 cm size), placed on aluminum stubs with double-sided conductive carbon tape, and coated with gold for 45 s using a Quorum SC7620 sputter coater (Quorum Technologies Ltd., Lewes, UK) to improve conductivity. Samples were then observed at 300× magnification under an accelerating voltage of 5 kV [39].

## 3. Results and Discussion

### 3.1. Response Surface Methodology (RSM) Optimization of Kai Food Ink

After preparation and printing of the 16 formulations generated from the mixture optimal design, the measured responses were analyzed using RSM to evaluate the effects of the blend proportions of XG and KG on the textural and structural properties of Kai food inks. RSM is an effective statistical tool for optimizing multi-variable food systems [40] and 3D-printable food inks [36]. The responses included hardness, adhesiveness, cohesiveness, syneresis, print accuracy, and deformation, which collectively describe the printability and structural stability of the formulations. The experimental results of all formulations are presented in Table 3 and were subsequently used for model development and numerical optimization.

### 3.2. Variation in Measured Responses Across Formulations

The experimental data showed considerable variation among the investigated formulations, indicating that the structural, physical, and handling properties of the Kai algae food matrix were strongly influenced by the hydrocolloid composition. As presented in Table 4, hardness exhibited the highest variability within the design space, ranging from 604.23 N/m^2^ (Run 2: 0% XG, 9% KG) to 3310.52 N/m^2^ (Run 4: 6.91% XG, 2.09% KG). This wide distribution (SD = 706.12 N/m^2^) demonstrates that the mechanical strength of the printed samples could be adjusted by varying the blend proportions of XG and KG. Formulations containing balanced blend proportions or XG-rich compositions generally produced stronger and more self-supporting structures than formulations dominated by KG or containing lower hydrocolloid levels [13,41].

In contrast, print accuracy remained relatively consistent across all formulations, ranging from 91.87% to 96.10%, with a low standard deviation of 1.13%. The high print accuracy indicates that the printed structures closely matched the dimensions of the designed model, reflecting good shape retention after extrusion. This suggests that the combination of finely sieved tray-dried Kai powder and hydrocolloid hydration provided stable flow behavior and sufficient structural support during printing, enabling uniform deposition through the 1.5 mm nozzle while minimizing defects such as spreading, filament collapse, or nozzle blockage [42].

Syneresis values remained within a narrow range from 1.54% to 2.84%, with a standard deviation of 0.35%, indicating good water retention stability among the formulations. This suggests that the hydrocolloid network effectively immobilized water within the matrix and minimizes phase separation during and after printing. This characteristic is desirable for 3D food printing because excessive water separation can adversely affect dimensional stability, texture, and product quality during storage and handling.

Deformation values ranged from 0.07% to 2.39%, showing clear differences in structural stability after printing. Formulations containing balanced blend proportions of XG and KG, particularly Runs 11 and 14, exhibited very low deformation values (<0.20%), indicating improved shape retention and structural stability. This behavior suggests synergistic viscoelastic interactions between the two hydrocolloids, where XG contributed shear-thinning behavior and KG enhanced elastic recovery and structural reinforcement [21]. Overall, the observed variation in the measured responses confirms that the blend proportions of XG and KG strongly influenced the textural and structural properties of the Kai food inks, providing the basis for subsequent response surface analysis and optimization.

### 3.3. Model Fitting and Statistical Adequacy

To evaluate the relationship between XG: *x*_1_, KG: *x*_2_, and the measured responses of the Kai algae food ink, regression models were developed using RSM. The regression coefficients and model-fitting statistics, including *R*^2^, Adjusted *R*^2^, Predicted *R*^2^, Adequate Precision, *F*-values, *p*-values, degrees of freedom, and lack-of-fit statistics, are presented in Table 5. All six developed models were statistically significant (*p* ≤ 0.0012, F 17.26 to 58.51), with *R*^2^ values ranging from 0.8456 to 0.9750. Hardness, syneresis, cohesiveness, and deformation models explained more than 93% of the variability in the experimental data, while the print accuracy model showed a comparatively lower but still substantial *R*^2^ value of 0.8456 (*R*^2^ = 0.8456) [42].

The reliability of the models was further supported by the close agreement between Adjusted *R*^2^ and Predicted *R*^2^ values, where all differences remained below 0.20. This indicates reasonable predictive performance within the experimental design space. In addition, Adequate Precision values for all responses were higher than the recommended minimum value of 4.00, ranging from 12.63 for syneresis to 27.09 for hardness. These results indicate that the generated models had sufficient signal-to-noise ratios and could be used to predict and navigate the experimental design space effectively [13,43]. The lack-of-fit test was non-significant for adhesiveness, cohesiveness, syneresis, and deformation (*p* > 0.05), but significant for hardness (*p* = 0.0256) and print accuracy (*p* = 0.0015). Nevertheless, the corresponding models were statistically significant (*p* < 0.0001), with *R*^2^ values of 0.9750 and 0.8456, respectively. The selected models had 3–9 model degrees of freedom, with 5 degrees of freedom for pure error provided by the five replicate pairs. Among the selected models, the cubic syneresis model had the highest complexity, with 9 model degrees of freedom and 6 residual degrees of freedom. The syneresis model was statistically significant (*p* = 0.0012), while its lack-of-fit was non-significant (*p* = 0.9516). These statistics provide information on the model complexity and available degrees of freedom for assessing the higher-order model. Overall, the fitted models provided a basis for describing the relationships between hydrocolloid blend proportions and the measured responses and for subsequent numerical optimization.

Based on the regression coefficients shown in Table 5, the predictive equations for the measured responses were generated as follows:

Hardness (N/m^2^) = 582.1 + 133.3*x*_1_ + 237.9*x*_2_ − 86.84*x*_1_*x*_2_
*−* 8.971*x*_1_^2^ − 25.53*x*_2_^2^ + 30.36*x*_1_^2^*x*_2_ (4)

Adhesiveness (J/m^3^) = 126.3 − 5.39*x*_1_ + 10.32*x*_2_ + 3.821*x*_1_^2^ − 0.3488*x*_2_^2^ − 0.3084*x*_1_^3^ (5)

Cohesiveness = 0.4062 + 0.0083*x*_1_
*−* 0.01566*x*_2_ + 0.01909*x*_2_^2^
*−* 0.001701*x*_2_^3^ (6)

Print Accuracy (%) = 90.68 + 0.4128*x*_1_ + 0.9251*x*_2_ − 0.06136*x*_2_^2^ (7)

Syneresis (%) = −15.35 + 11.13*x*_1_ + 10.978*x*_2_ − 3.941*x*_1_*x*_2_ − 2.118*x*_1_^2^ − 1.993*x*_2_^2^ + 0.3379*x*_1_^2^*x*_2_ + 0.3225*x*_1_*x*_2_^2^+ 0.1214*x*_1_^3^ + 0.1101*x*_2_^3^ (8)

Deformation (%) = 3.406 − 0.3379*x*_1_ − 0.6094*x*_2_ + 0.0465*x_2_*^2^ (9)

Because XG and KG were individually varied from 0 to 9%, while their total concentration was constrained to 3–9%, the regression equations (Equations (4)–(9)) describe the response behavior across the XG–KG combinations evaluated under these constraints. Therefore, the regression coefficients of *x*_1_ and *x*_2_ should be interpreted within the tested XG–KG combinations rather than as completely independent effects across an unrestricted factor space. The interaction, linear, quadratic, and cubic terms indicate that the responses were influenced by the individual concentrations of XG and KG, their interactions, and nonlinear effects. Consequently, the response patterns observed in the contour plots reflect changes in both XG–KG blend proportion and total hydrocolloid concentration. Overall, these results demonstrate that the textural, structural, and printing properties of the Kai algae food ink were associated with the combined effects of XG and KG, providing a basis for subsequent response surface analysis and numerical optimization.

### 3.4. Evaluation of Individual Responses & Response Surface Analysis

To evaluate the interactive effects of XG and KG on the structural properties of the Kai algae food ink, two-dimensional contour plots were analyzed for each measured response. These plots illustrate how changes in hydrocolloid composition influenced the textural and structural characteristics of the printed formulations.

#### 3.4.1. Interactive Effects on Textural Attributes

The texture profile of the printed formulations is important for both structural stability and dysphagia suitability. The contour plots for hardness, adhesiveness, and cohesiveness are discussed below to explain the effects of XG and KG interactions within the formulation system.

##### Hardness

**Figure 3:**
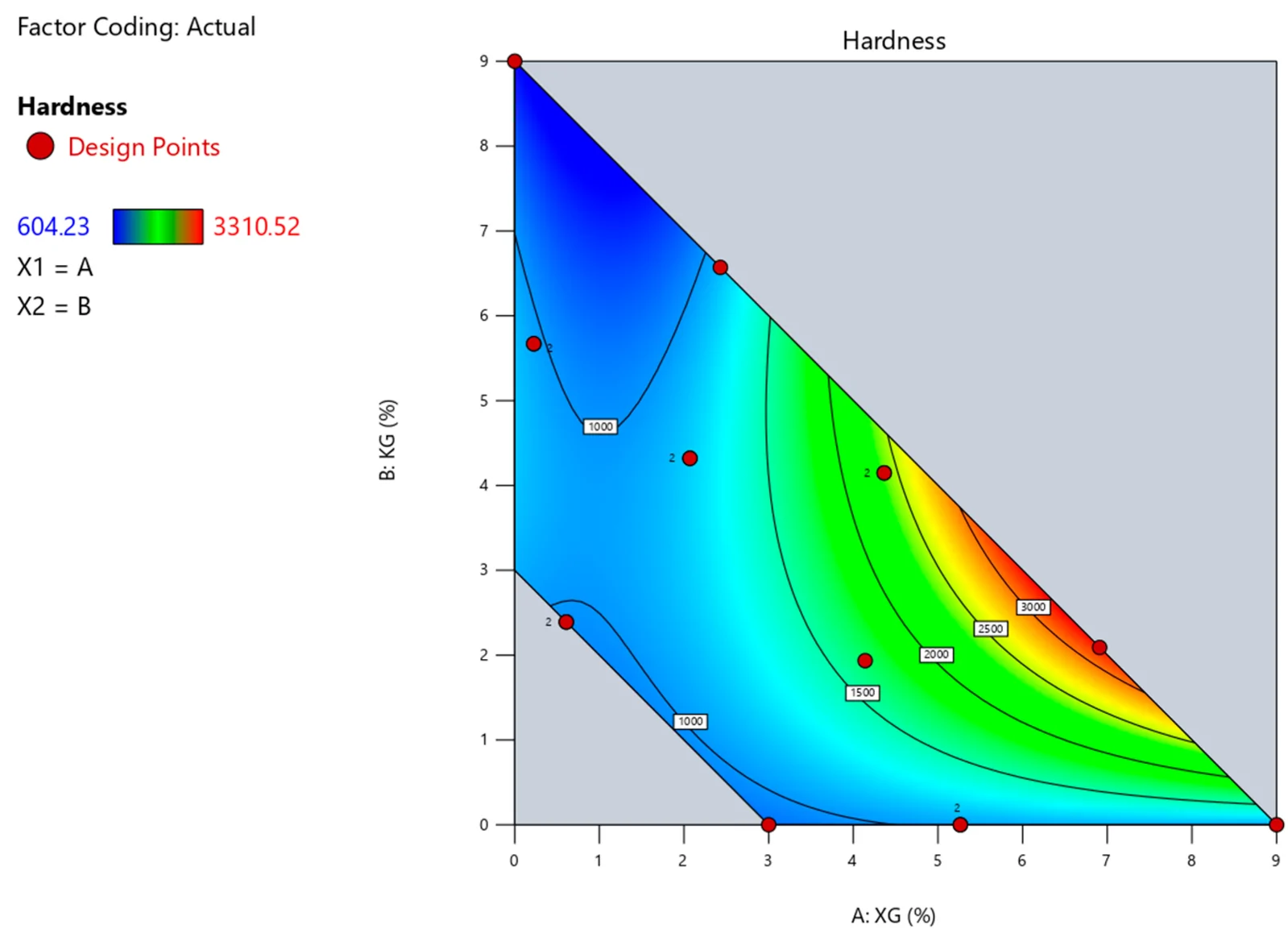
Contour plot showing the interactive effects of xanthan gum (XG) and konjac gum (KG) concentrations on hardness of 3D-printed Kai algae food inks.

The hardness of the printed Kai algae formulations was significantly influenced by the proportions of XG and KG and was adequately described by a special cubic model, ranging from 604.23 to 3310.52 (Figure 3). The contour plot demonstrated that hardness was strongly influenced by the proportions of XG and KG in the formulation. Higher hardness values were mainly observed at elevated XG concentrations (6–7%) combined with low-to-moderate KG concentrations (2~3%). This suggests that formulations containing higher proportions of XG and moderate proportions of KG exhibited greater mechanical strength. The observed response indicates that hardness was influenced by the combined effects of both hydrocolloids, with the XG ratio playing an important role in determining the structural properties of the printed matrix [8,17].

In contrast, formulations containing high KG concentrations and low XG levels exhibited lower hardness values. The curved contour lines indicate a non-linear relationship between hydrocolloid composition and hardness, suggesting that the effect of each component depended on the proportion of the other component. Furthermore, hardness tended to decrease at higher KG concentrations, indicating that increasing KG beyond a certain level was not beneficial for hardness development. Similar non-linear responses have been reported in hydrocolloid systems, where the functional properties depend on the balance and interactions between polymers rather than their individual concentrations alone [21,44]. These findings indicate that optimizing the blend proportions of XG and KG is important for achieving sufficient mechanical strength to maintain the structural integrity of the printed Kai algae food during and after extrusion.

##### Cohesiveness

**Figure 4:**
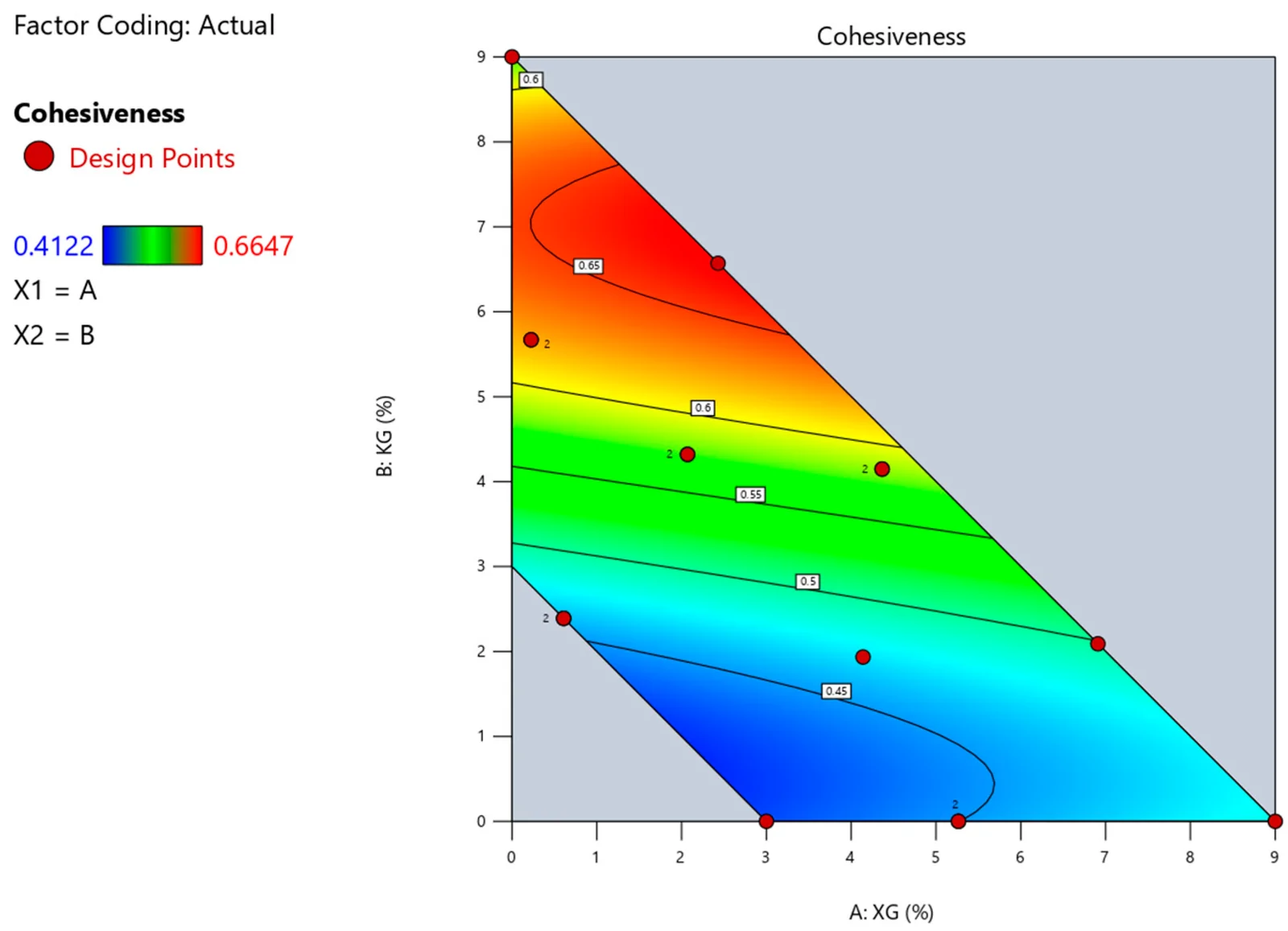
Contour plot showing the interactive effects of XG and KG concentrations on cohesiveness of 3D-printed Kai algae food inks.

Cohesiveness, which represents the internal bonding strength and resistance to structural breakdown during mastication, showed a third-order cubic relationship with values ranging from 0.4122 to 0.6647 (Figure 4). The contour plot indicated that cohesiveness was strongly influenced by the relative proportions of XG and KG in the formulation. Higher cohesiveness values were observed in formulations containing higher proportions of KG, suggesting that KG had a greater contribution to enhancing the internal structural integrity of the printed matrix. This observation may be attributed to the high water-binding capacity and gel-forming ability of KG, which promotes the formation of a stronger hydrated polymer network and improves structural integrity [22,45,46].

In comparison, changes in XG concentration produced relatively smaller effects on cohesiveness, suggesting that XG primarily contributed to viscosity enhancement and extrusion stability, while KG had a greater influence on internal gel strength [16,19]. The contour lines also showed that formulations with low KG concentrations exhibited the lowest cohesiveness values, particularly in regions containing high XG and minimal KG.

Balanced combinations of XG and KG at intermediate concentrations produced moderate cohesiveness values without excessively high stickiness. This suggests that combining the two hydrocolloids contributes to improved structural balance and suitable bolus formation, which are desirable characteristics for dysphagia-oriented food applications [13].

##### Adhesiveness

**Figure 5:**
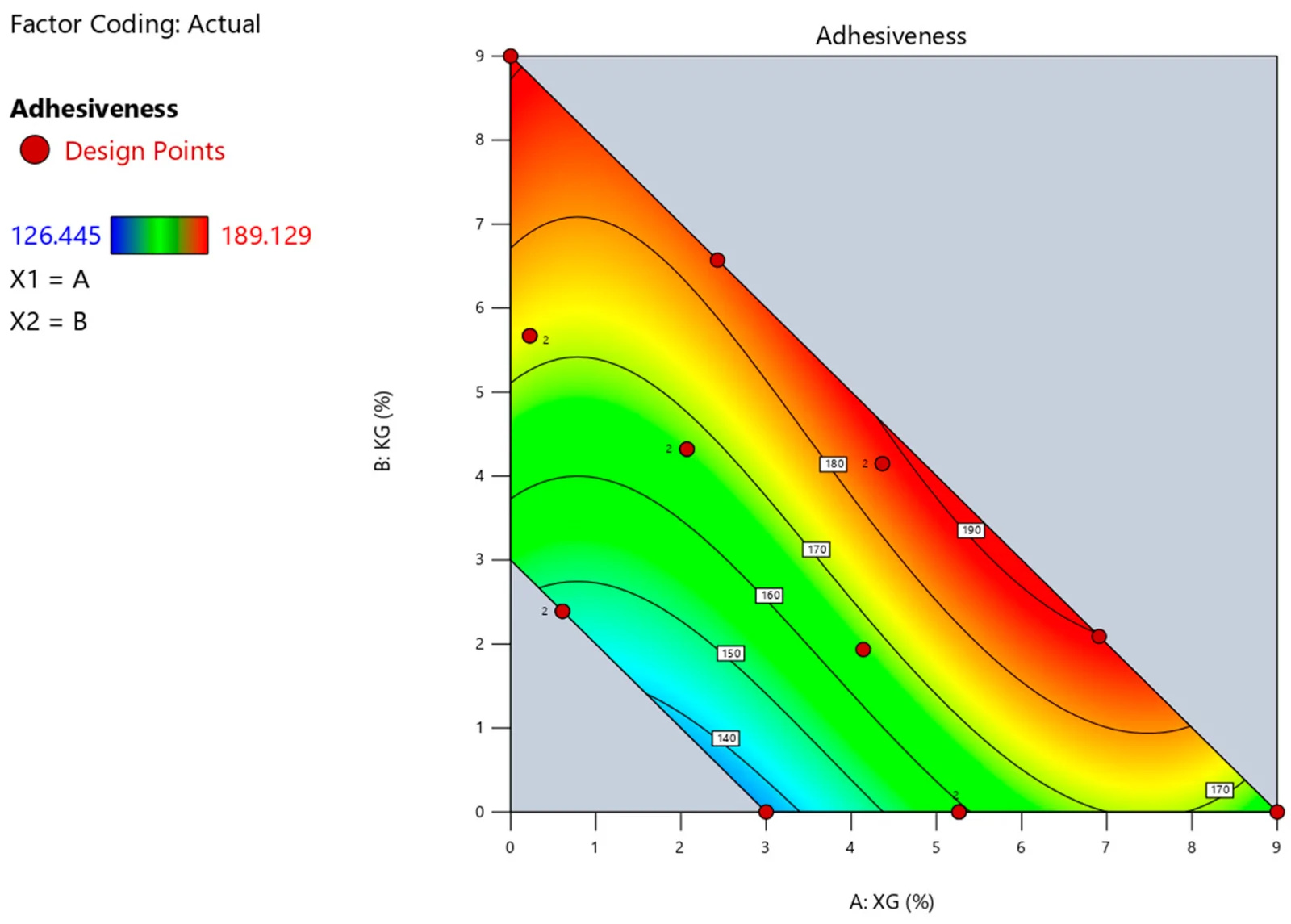
Contour plot showing the interactive effects of XG and KG concentrations on adhesiveness of 3D-printed Kai algae food inks.

Adhesiveness, which represents the work required to overcome the attractive forces between the food surface and another material during oral processing, ranged from 126.26 to 189.13 J/m^3^ and showed a curved response pattern across the design space (Figure 5) [47]. The contour plot demonstrated that adhesiveness was strongly influenced by the relative proportions of XG and KG in the formulation. Higher adhesiveness values were generally observed in formulations with total gum (>7%) containing high and moderate proportions of KG, suggesting that KG had a greater influence on the development of surface stickiness. This observation may be associated with the high water-binding capacity and hydrophilic nature of KG, which promotes hydration of the polymer chains and increases surface adhesion in hydrocolloid-based systems [8,46].

In comparison, changes in the proportion of XG resulted in relatively smaller variations in adhesiveness. This may indicate that XG primarily influenced bulk viscosity and flow characteristics rather than surface adhesion. The contour lines indicated that formulations containing moderate-to-high proportions of KG exhibited the highest adhesiveness values, whereas formulations with lower KG proportions showed reduced surface stickiness. Furthermore, formulations containing balanced proportions of XG and KG exhibited moderate adhesiveness values, indicating that the response depended on the relative proportions of the two hydrocolloids rather than on either component alone [19,21].

This reduction may be attributed to possible synergistic interactions between XG and KG, which may promote the formation of a more integrated gel network and limit the availability of surface hydrophilic groups [44,48]. Controlled adhesiveness is important for dysphagia-oriented foods because excessive stickiness may increase the risk of food residues remaining in the oral or pharyngeal cavity after swallowing [13,49].

#### 3.4.2. Interactive Effects on Printability

The structural retention and processing fidelity of printed Kai constructs are governed by the balance between the ink’s shear-thinning behavior and its mechanical yield stress after extrusion. Print accuracy and deformation displayed highly significant quadratic relationships, demonstrating sharp sensitivity to the XG-KG blending ratio.

##### Print Accuracy

**Figure 6:**
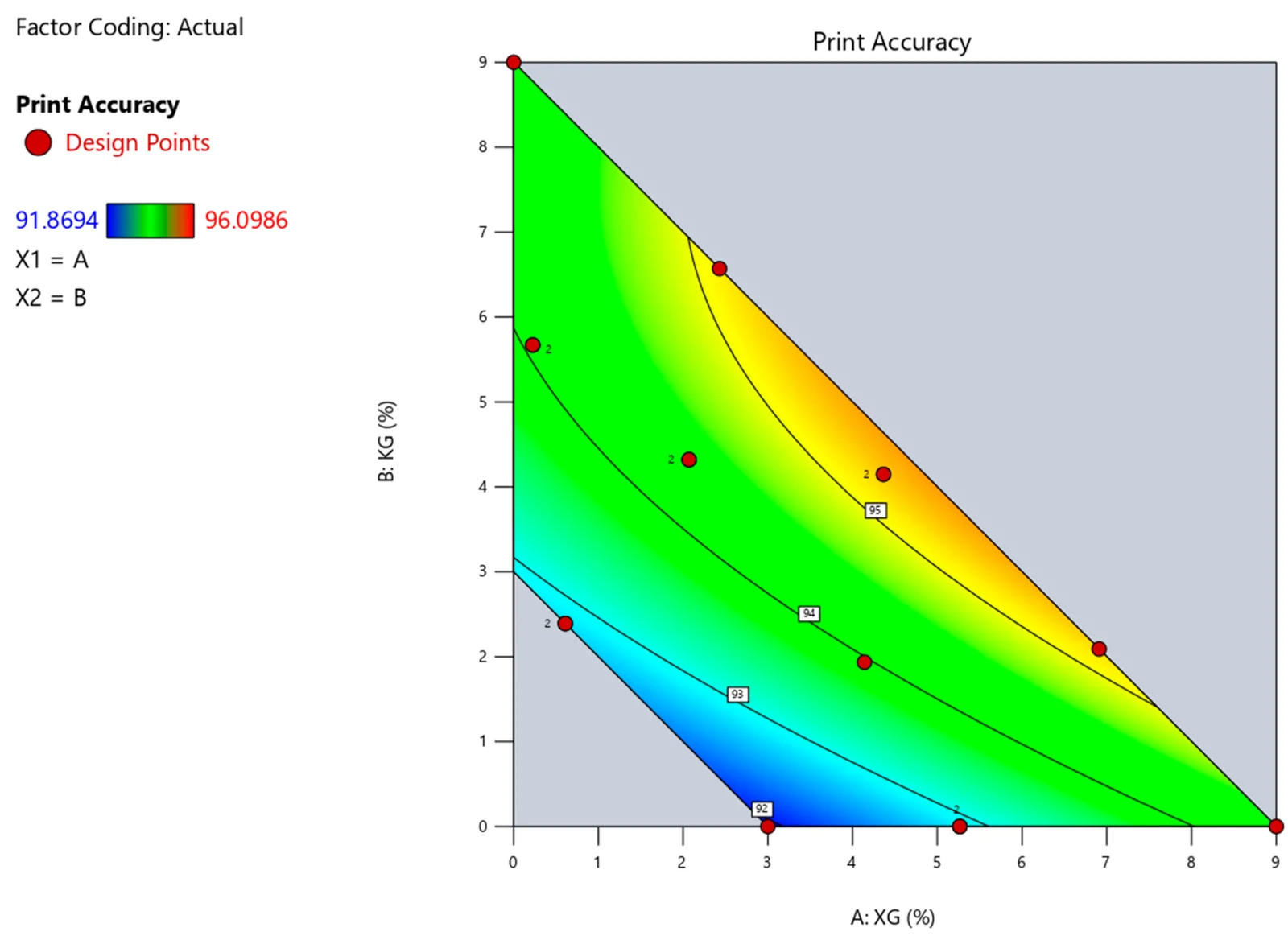
Contour plot showing the interactive effects of xanthan gum XG and KG concentrations on print accuracy of 3D-printed Kai algae food inks.

Print accuracy, which represents the degree of agreement between the dimensions of the printed structure and the designed model, ranged from 91.87% to 96.10% and was influenced by the combined concentrations of XG and KG (Figure 6) [50]. These values indicate good agreement between the printed structures and the intended design geometry. Although no universal print accuracy threshold has been established, dimensional quality criteria summarized by Čukelj Mustač et al. (2023) [43] suggest that satisfactory printing performance can be achieved when width consistency is maintained within ±5% of the designed width, printed line diameter does not exceed 130% of the nozzle diameter, and die swell remains below 70%.

The contour plot showed that higher print accuracy values were achieved at intermediate-to-high concentrations of both hydrocolloids, particularly in balanced mixed-gum regions. These formulations produced more defined and stable printed structures, indicating improved extrusion behavior yield stress and shape retention after deposition. Extrusion-based 3D printing requires a pseudoplastic fluid that undergoes rapid shear-thinning inside the nozzle for smooth flow, followed by a rapid recovery of viscosity and yield stress upon deposition to support successive layers [24,51]. A comprehensive review on 3D-printed dysphagia foods highlighted XG and KG have been reported as effective hydrocolloids for improving rheological properties and enabling nutrient-rich foods to achieve IDDSI compliance [52]. Tracing the contour lines down to low total concentration regimes shows a drastic drop in accuracy. When total biopolymer levels are insufficient, the weakened network structure cannot sufficiently resist deformation after extrusion, causing the deposited strands to spread excessively and reducing geometric fidelity [53,54].

##### Deformation

**Figure 7:**
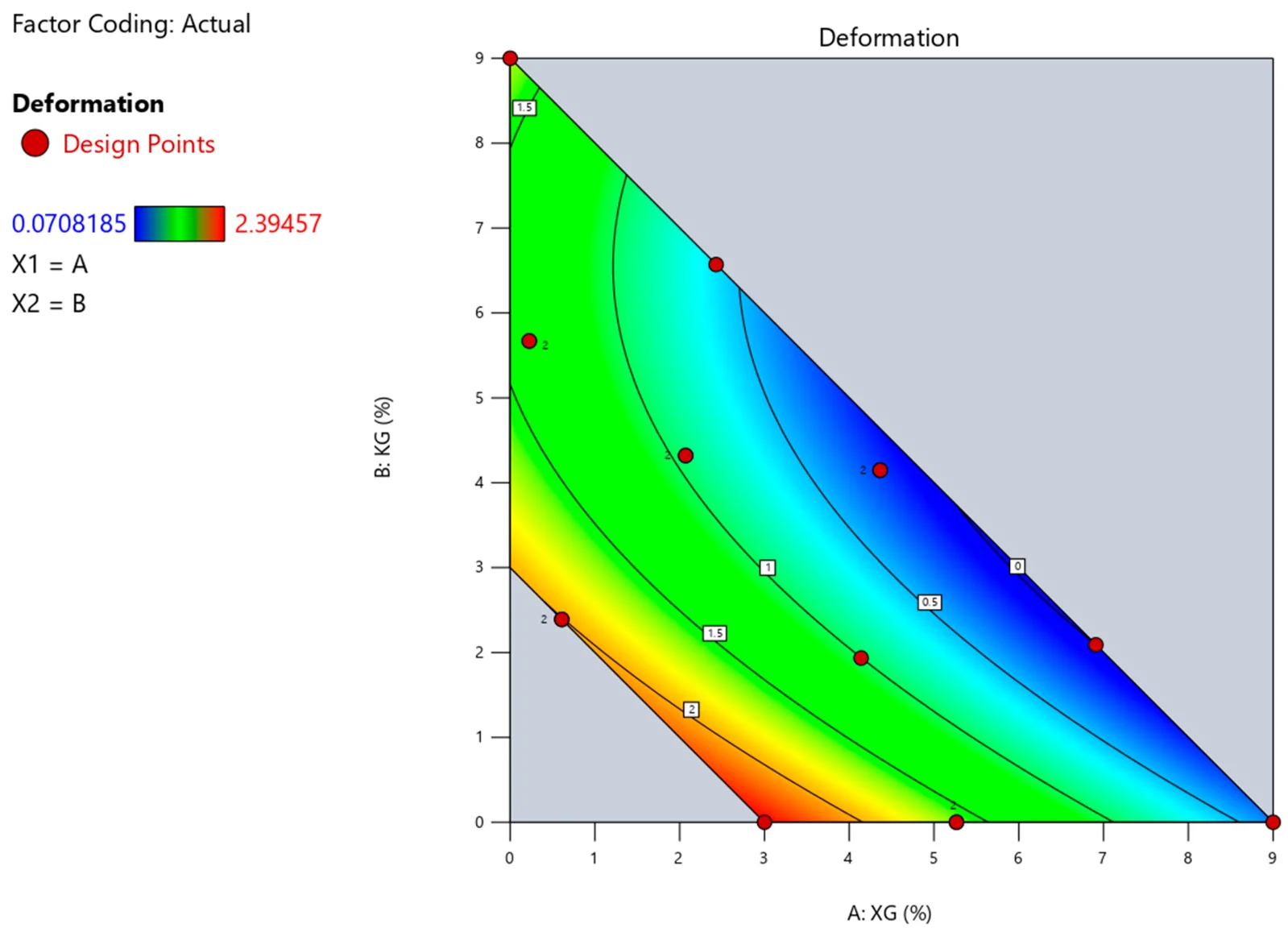
Contour plot showing the interactive effects of XG and KG concentrations on deformation of 3D-printed Kai algae food inks.

Deformation, which represents the dimensional change of the printed structure after deposition, ranged from 0.07% to 2.39%, indicating variations in the dimensional stability of the printed formulations after printing (Figure 7) [42,55]. The contour plot showed that the lowest deformation values were observed at high XG concentrations combined with low-to-moderate KG concentrations, particularly in the region around 5–8% XG and 1–3% KG. These formulations exhibited better shape retention and structural stability after printing.

In contrast, higher deformation values were mainly observed in formulations containing low XG concentrations with either very low or very high KG concentrations. This suggests that insufficient XG concentration weakened the structural framework of the printed formulations, resulting in greater deformation after deposition.

The reduced deformation observed in balanced XG–KG systems may be associated with the combined effects of the two hydrocolloids, where XG contributes to shear-thinning behavior during extrusion, while KG enhances gel network formation and structural reinforcement after deposition [16,22,56]. Extrusion-based 3D printing requires sufficient yield stress and viscoelastic properties to maintain shape fidelity after deposition [15]. These findings were consistent with the rheological results, where formulations exhibiting lower *n*, higher *K*, and greater yield stress demonstrated improved printability and shape retention [21].

#### 3.4.3. Interactive Effects on Syneresis

**Figure 8:**
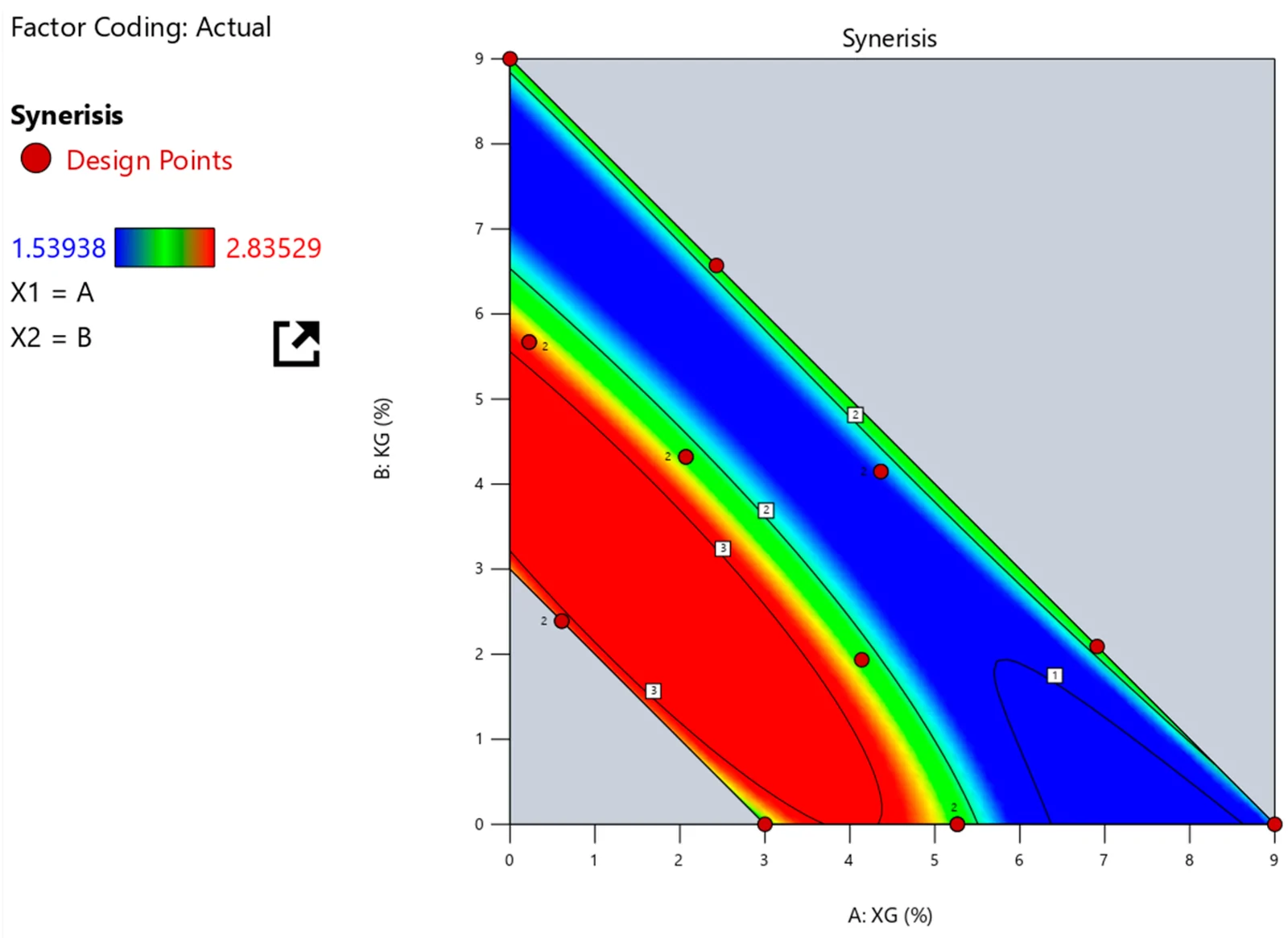
Contour plot showing the interactive effects of XG and KG concentrations on syneresis of 3D-printed Kai algae food inks.

Syneresis, reflecting water separation from the hydrocolloid matrix, was assessed based on the diffusion area of released liquid and ranged from 1.54 to 2.84 cm^2^ (Figure 8). Overall, all formulations exhibited relatively small diffusion areas, indicating limited water separation and good phase stability within the Kai food ink system.

The contour plot showed that lower syneresis values were mainly observed at moderate-to-high concentrations of both XG and KG, particularly in balanced mixed-gum regions. In contrast, formulations containing low XG concentrations with intermediate KG levels exhibited relatively higher syneresis values. These findings suggest that the interaction between XG and KG improved water retention within the hydrocolloid matrix. The improved stability may be associated with the ability of XG to increase viscosity and restrict water separation, while KG contributes to the formation of a three-dimensional gel network that may enhance water retention through polymer–water interactions [17,18,44].

The contour plot also indicated that the reduction in syneresis became less pronounced at very high hydrocolloid concentrations, suggesting limited additional improvement in water retention at elevated gum levels. This behavior may be related to reduced availability of free water and possible limitations in further network development at excessive hydrocolloid concentrations [21]. The diffusion area measured by the filter-paper method provides an indirect indication of water separation from the hydrocolloid matrix. Compared with centrifugation-based methods, which allow more direct quantification of separated liquid, the measured diffusion area may also be influenced by sample spreading, filter-paper absorption, and evaporation. Under standardized conditions, these measurements provide a basis for comparing water separation among the different Kai food ink formulations.

### 3.5. Numerical Multi-Response Optimization

Multi-response optimization was conducted to determine the optimum blending ratio of XG (*x*_1_) and KG (*x*_2_) using a desirability function approach [57]. The optimization aimed to develop a formulation that balanced printability, structural stability, and texture properties suitable for dysphagia-oriented food applications. By simultaneously evaluating all measured responses through the generated mathematical models as commonly applied in food formulation optimization using response surface methodology, the optimization process identified a formulation region capable of achieving an appropriate balance between structural integrity, printability, deformation, and adhesiveness after printing [58].

**Figure 9:**
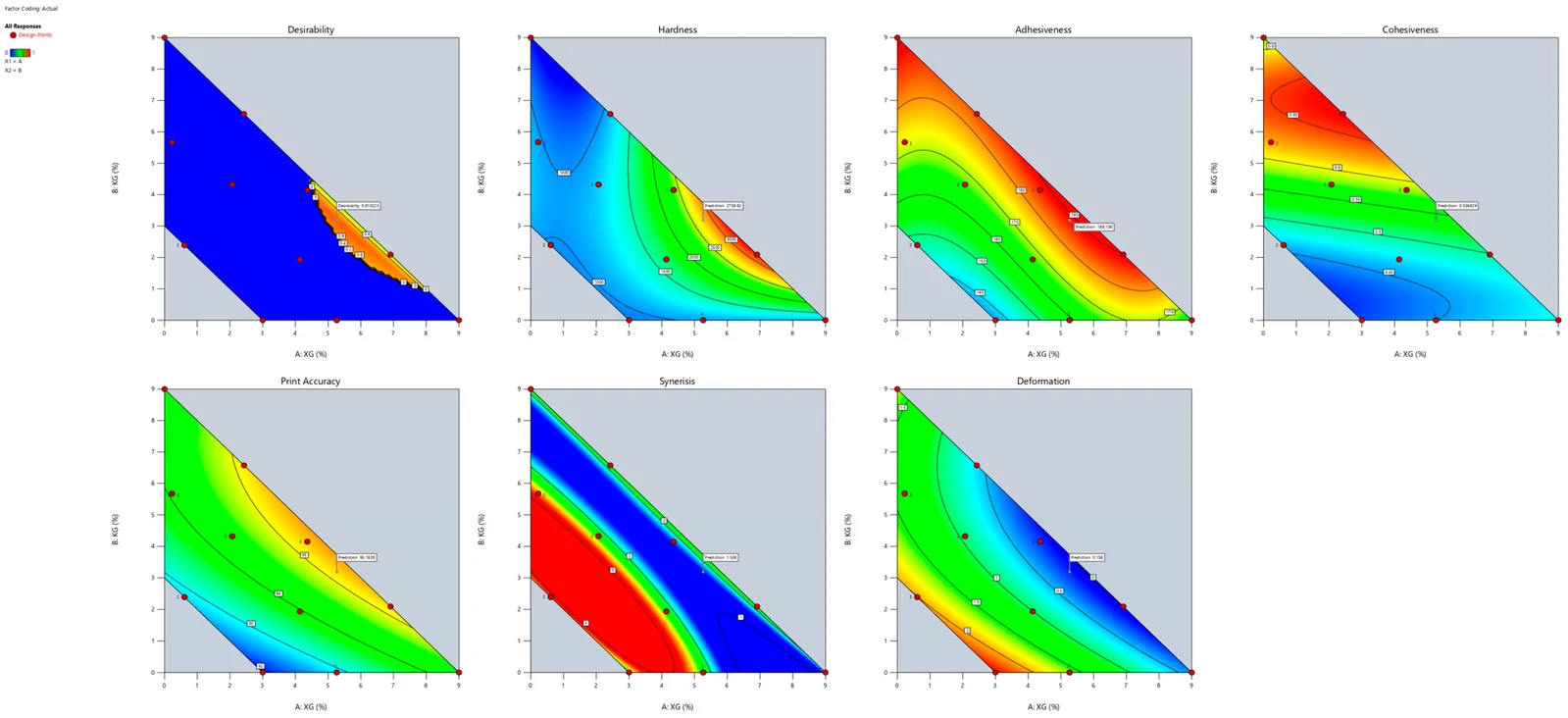
Contour plots showing the predicted responses and desirability at the predicted optimum XG–KG formulation.

The optimization identified a model-predicted optimum formulation with a maximum desirability value (*D* = 0.91) at a combination of 5.263% XG and 3.188% KG (Figure 9). The predicted response values represent model-based estimates within the experimental design space and require further experimental confirmation. This optimized formulation provided the most desirable balance between printability, structural stability, and dysphagia-oriented texture properties among the developed formulations. The formulation exhibited high predicted print accuracy (95.18%) together with a low predicted deformation value (0.157%), indicating good shape retention and post-print structural stability.

Under the optimized conditions, the predicted hardness, adhesiveness, and cohesiveness values were 2758 N/m^2^, 188.1 J/m^3^, and 0.539, respectively. These predicted values satisfied the Japanese Dysphagia Diet 2021 Criteria I and UDF Stage 4 requirements, indicating potential suitability as a texture-modified food for individuals requiring dysphagia management. Similarly, Zhu et al. reported that pea protein-based dysphagia diets containing 0.5% XG exhibited optimal 3D printing behavior and achieved characteristics corresponding to IDDSI Level 4 classification, supporting the effectiveness of hydrocolloid addition for texture-modified food development [59]. The optimized formulation also demonstrated favorable predicted stability characteristics, including low syneresis (1.539 cm^2^) and favorable structural stability, suggesting that the XG–KG hydrocolloid system provided suitable structural and textural properties for dysphagia-oriented food applications.

### 3.6. Visual Appearance of Printed Constructs

**Figure 10:**
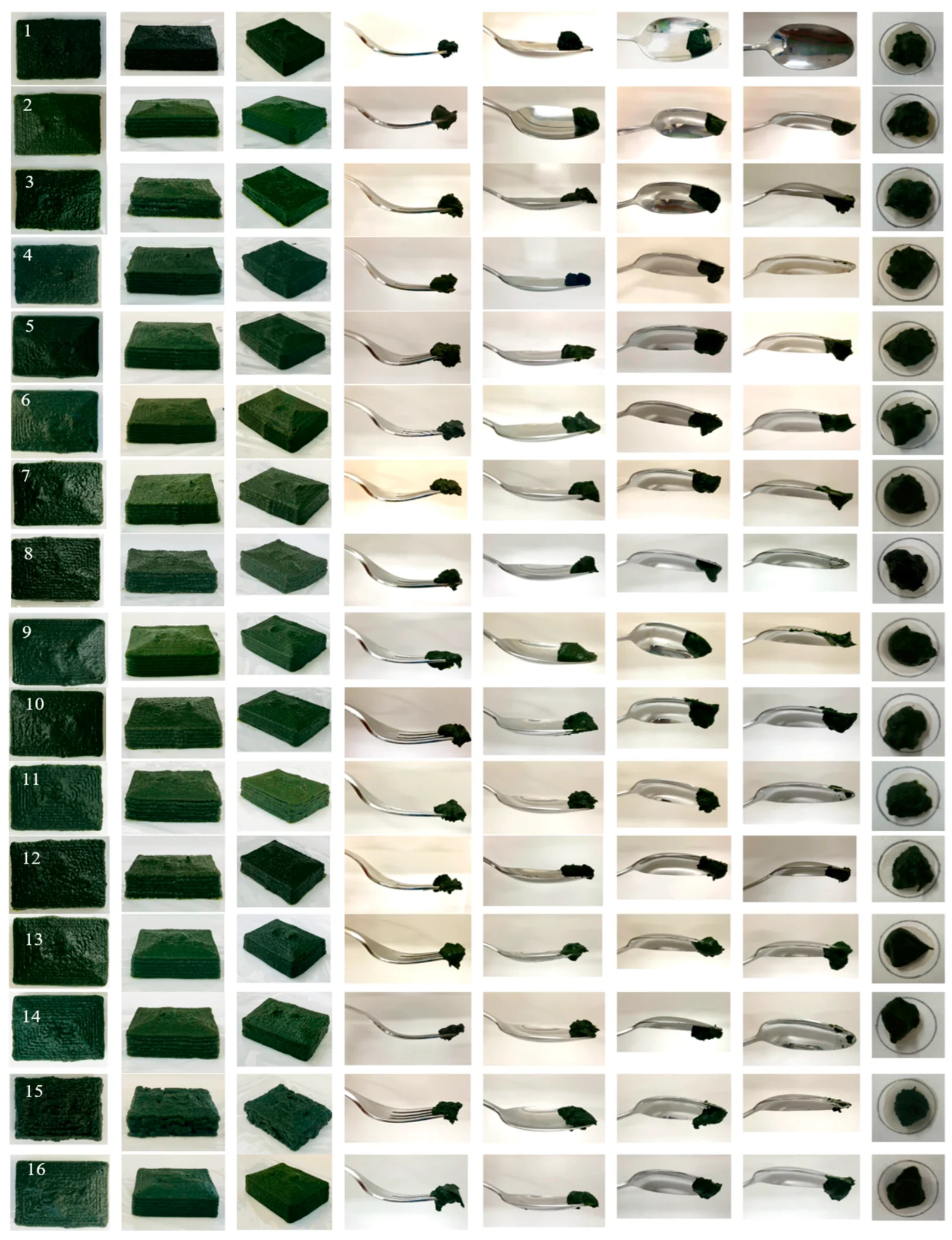
Representative images of 3D-printed Kai-based samples (Runs 1–16) with varying concentrations of XG and KG. Columns 1–3 show sample appearance, column 4 shows the IDDSI fork drip test, columns 5–7 show the IDDSI spoon tilt test, and column 8 shows the syneresis test.

Representative images of the printed Kai formulations are presented in Figure 10 to visually assess the post-print appearance and structural retention of samples prepared with different XG and KG combinations. All formulations retained their overall printed structure immediately after extrusion, indicating that the hydrocolloid systems provided sufficient viscosity and internal network strength to support layer deposition while maintaining the overall printed structure.

Minor variations in dimensional retention and structural preservation were observed among formulations, suggesting that hydrocolloid concentration influenced post-print behavior. Certain formulations exhibited slight spreading or deformation after deposition, whereas others maintained their printed dimensions more effectively. These visual observations were generally consistent with the measured print accuracy and deformation values obtained during optimization, where formulations containing balanced XG-KG ratios demonstrated improved structural retention after printing.

No visible liquid separation was observed in any formulation, which was consistent with the low syneresis values obtained. Furthermore, all printed samples remained self-supporting under ambient conditions without major structural failure. Overall, the results indicated that the combined XG-KG hydrocolloid system provided suitable structural stability and dimensional retention for extrusion-based 3D printing of Kai-based dysphagia-oriented foods.

### 3.7. Integrated Dysphagia Evaluation Based on Japanese Texture Criteria, UDF Classification and IDDS Assessment

The 3D-printed Kai formulations were evaluated for dysphagia suitability integrating TPA with the Japanese texture criteria, UDF framework, and IDDSI Level 4 criteria (Table 6). Hardness, cohesiveness, and adhesiveness were used as the main textural parameters.

Run 4 showed the highest hardness (3310.52 N/m^2^) and was classified under Japanese texture standard I, indicating suitability for foods requiring a high level of texture modification. Most other formulations fell within Standards II and III. However, hardness alone did not fully represent swallowing behavior, as cohesiveness and adhesiveness also influenced bolus formation and utensil handling [11].

All formulations met the IDDSI Level 4 fork drip test requirement, demonstrating that the samples retained their structure on the fork and did not exhibit excessive flow. Spoon test performance varied depending on internal gel structure [1]. Low-strength formulations (Runs 2, 3, 5, 6, 12, 13) spread on the spoon due to insufficient structural integrity. High-cohesiveness formulations (Runs 7, 9, 16) showed stronger adhesion to the spoon, which may reduce ease of release during consumption. Run 15 also passed both fork and spoon tests; however, its application was limited by poor surface uniformity and reduced print accuracy, indicating that its limitation was related to printing performance rather than swallowing functionality.

Based on the UDF classification criteria, all formulations were categorized as Stage 4 according to their measured hardness value below 5 × 10^3^ N/m^2^ [8]. Overall, suitable dysphagia textures require a balance between hardness, cohesiveness, and adhesiveness to ensure proper bolus formation and spoon release. Run 4 showed the most balanced performance for severe dysphagia applications. Runs 1, 8, 10, 11, and 14 also demonstrated acceptable functional behavior. However, run 15 was excluded from final application due to poor print accuracy despite acceptable IDDSI performance.

### 3.8. Rheological Properties of Food Inks

**Figure 11:**
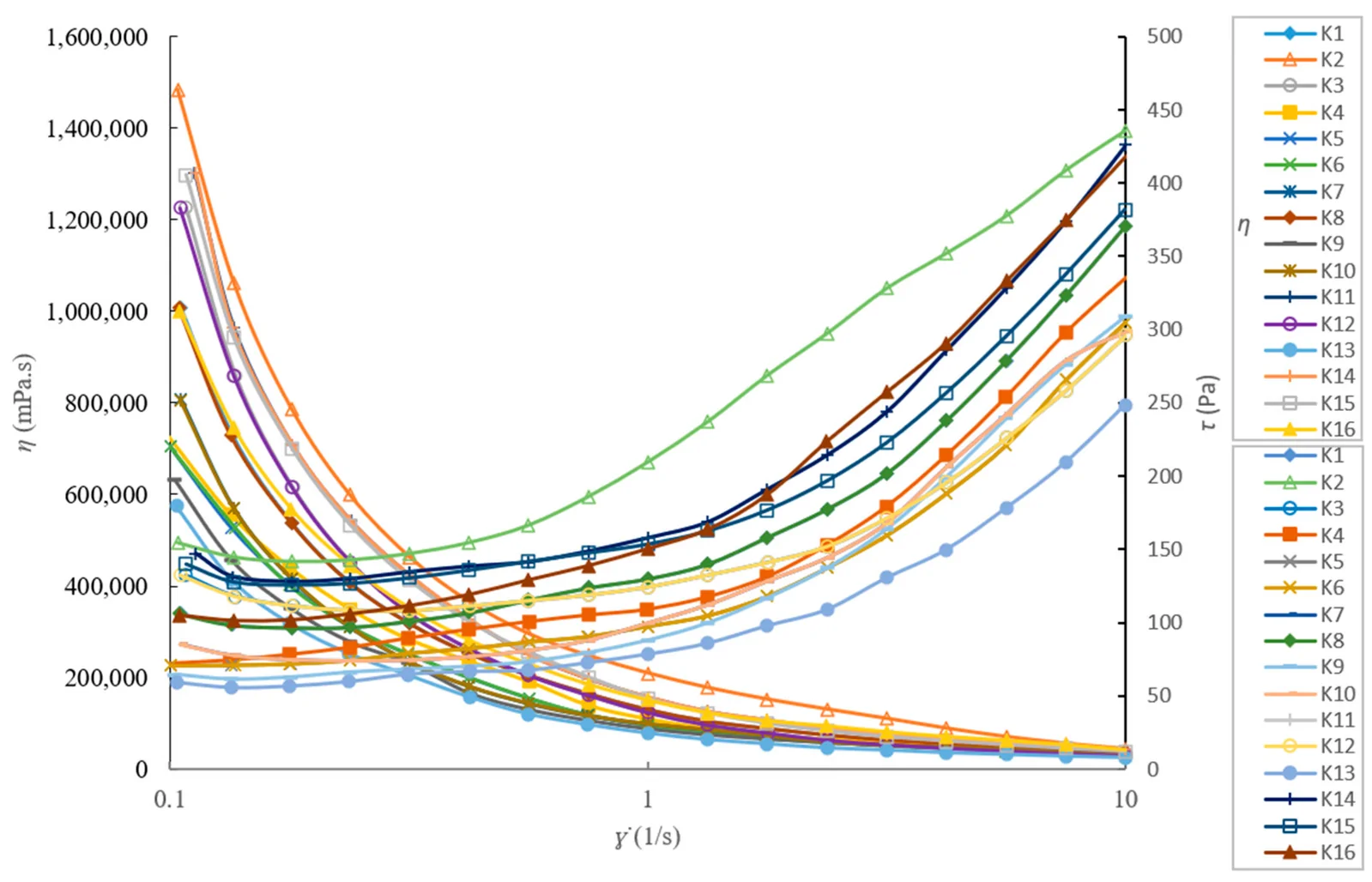
Apparent viscosity and shear stress as a function of shear rate of Kai-based food inks containing different ratios of XG and KG.

**Figure 12:**
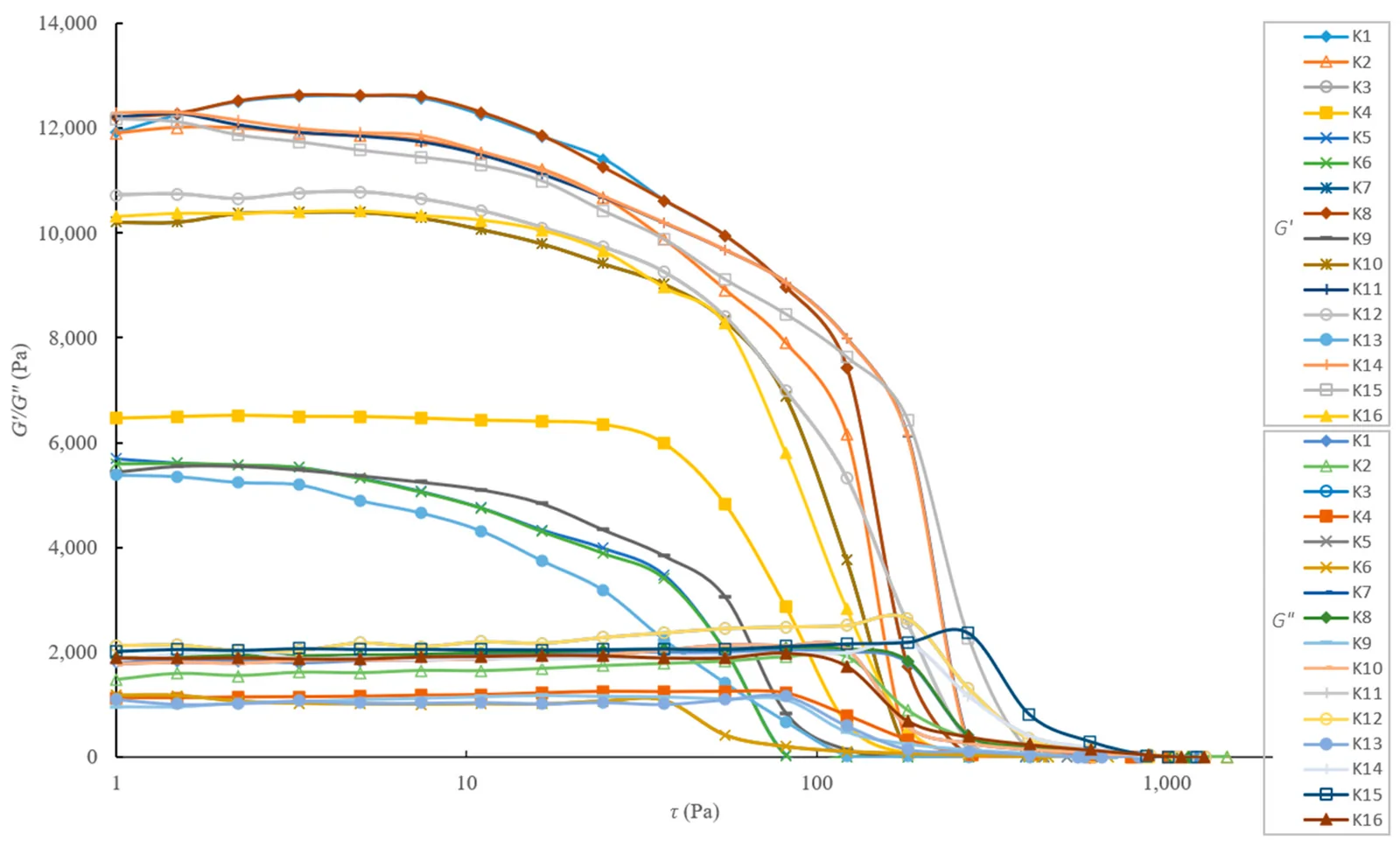
Stress sweep analysis of Kai-food inks with different XG and KG concentrations.

**Figure 13:**
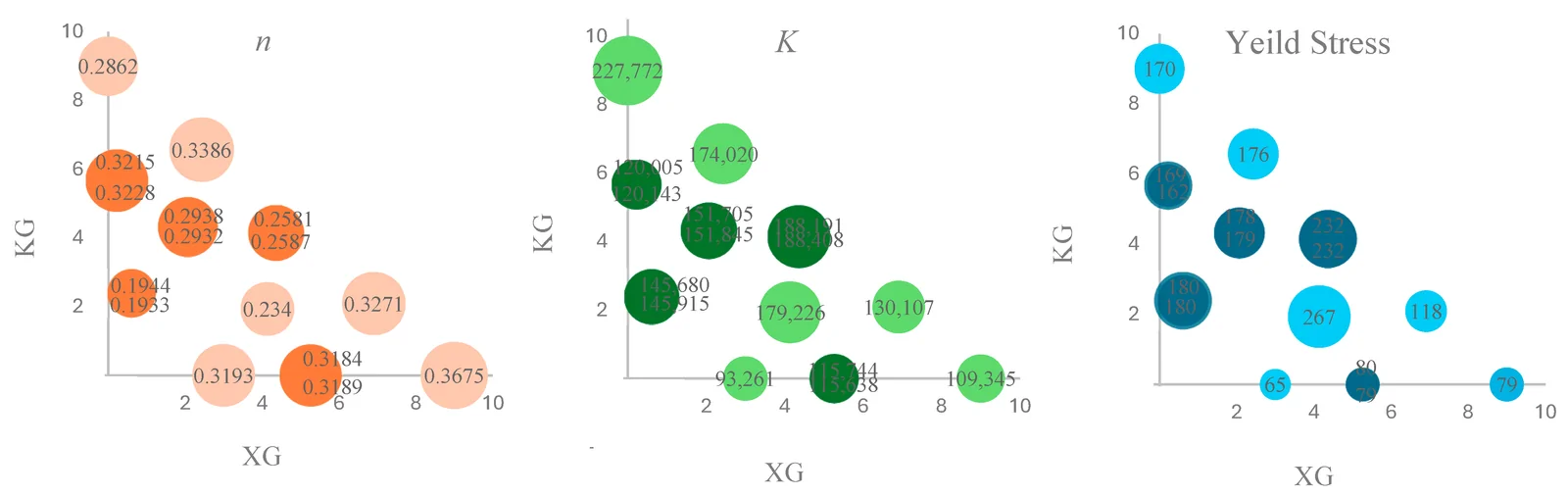
Effect of XG and KG on the rheological parameters of Kai-based food inks. Bubble size corresponds to the magnitude of the measured rheological parameter. Color intensity is used to differentiate replicated and non-replicated formulations in the optimal mixture design, with darker bubbles indicating replicated formulation points.

To further explain the differences observed in printability performance and structural stability of the Kai-based dysphagia formulations, rheological characterization was performed using steady shear and oscillatory stress sweep analyses. The rheological behaviors of formulations containing XG and KG are presented in Figure 11, Figure 12 and Figure 13.

As shown in Figure 11, all formulations exhibited decreasing viscosity with increasing shear rate, demonstrating typical pseudoplastic or shear-thinning behavior. Such behavior is desirable for extrusion-based dysphagia foods because the formulations can maintain structural integrity at low shear conditions while allowing easier flow during extrusion [16,21,60]. Differences in viscosity profiles among formulations suggested that the XG and KG influenced flow behavior of the food inks.

Stress sweep results are presented in Figure 12. In all formulations, *G*′ remained higher than *G*″ within the linear viscoelastic region, demonstrating predominantly elastic behavior [61,62]. With increasing applied stress, *G*′ gradually decreased until the crossover point between *G*′ and G″ was reached, representing structural breakdown and transition to viscous-dominant behavior. Mixed-gum systems generally maintained elastic behavior over a wider stress range than single-gum formulations, suggesting enhanced network stability under applied stress [44,63].

To further quantify the rheological properties, the steady shear data were fitted using the Power Law model [61,64]. The apparent viscosity (*η*) as a function of shear rate ($\overset{\cdot }{\gamma }$) can be described by the following relationship: $\eta =K\cdot {\overset{\cdot }{\gamma }}^{\left(n-1\right)}$. This form directly describes the shear-thinning behavior observed in Figure 11, where viscosity decreases with increasing shear rate when *n* < 1 [13,16]. The Power Law fitting parameters (*K* and *n*) and yield stress for all formulations are presented in Figure 13.

All formulations exhibited *n* values lower than 1 (0.1933–0.3675), confirming typical shear-thinning behavior expected for extrudable food gels [15,61]. The *R*^2^ values ranged from 0.9680 to 0.9892, indicating that the Power Law model provided an excellent fit to the experimental data across all formulations. Mixed XG-KG formulations generally exhibited higher *K* and yield stress values than single-gum systems, indicating increased flow resistance and structural strength. Formulations with moderate-to-high yield stress values (180–232 Pa) generally showed higher print accuracy (0.945–0.961) and lower post-print deformation (<0.9%), whereas formulations with very low yield stress values (65–80 Pa) exhibited lower print accuracy (0.918–0.931) and greater deformation (up to 2.4%). This observation agrees with previous studies reporting that sufficient yield stress is important for maintaining shape fidelity after extrusion [15,55]. However, the formulation with the highest yield stress value (267 Pa) did not exhibit the highest print accuracy, suggesting that excessive rigidity may impair extrusion stability. Similarly, formulations with the lowest *n* values (0.193–0.194) showed comparatively higher deformation, suggesting that excessive shear-thinning behavior alone was insufficient to maintain post-print stability.

Overall, balanced rheological behavior characterized by moderate-to-high yield stress and consistency together with adequate shear-thinning behavior generally provided improved print accuracy, lower post-print deformation, and firmer texture characteristics. In contrast, formulations with very low yield stress showed insufficient resistance to deformation, whereas excessively high yield stress reduced extrusion stability. Relationships between rheological behavior and cohesiveness, adhesiveness, or syneresis were less consistent, suggesting that these properties were additionally influenced by water distribution and formulation interactions within the matrix. These findings suggest that optimization of dysphagia-oriented food inks requires careful adjustment of the XG–KG ratio to achieve sufficient viscosity and structural resistance for shape fidelity without excessively restricting flow behavior.

### 3.9. Microstructure SEM

**Figure 14:**
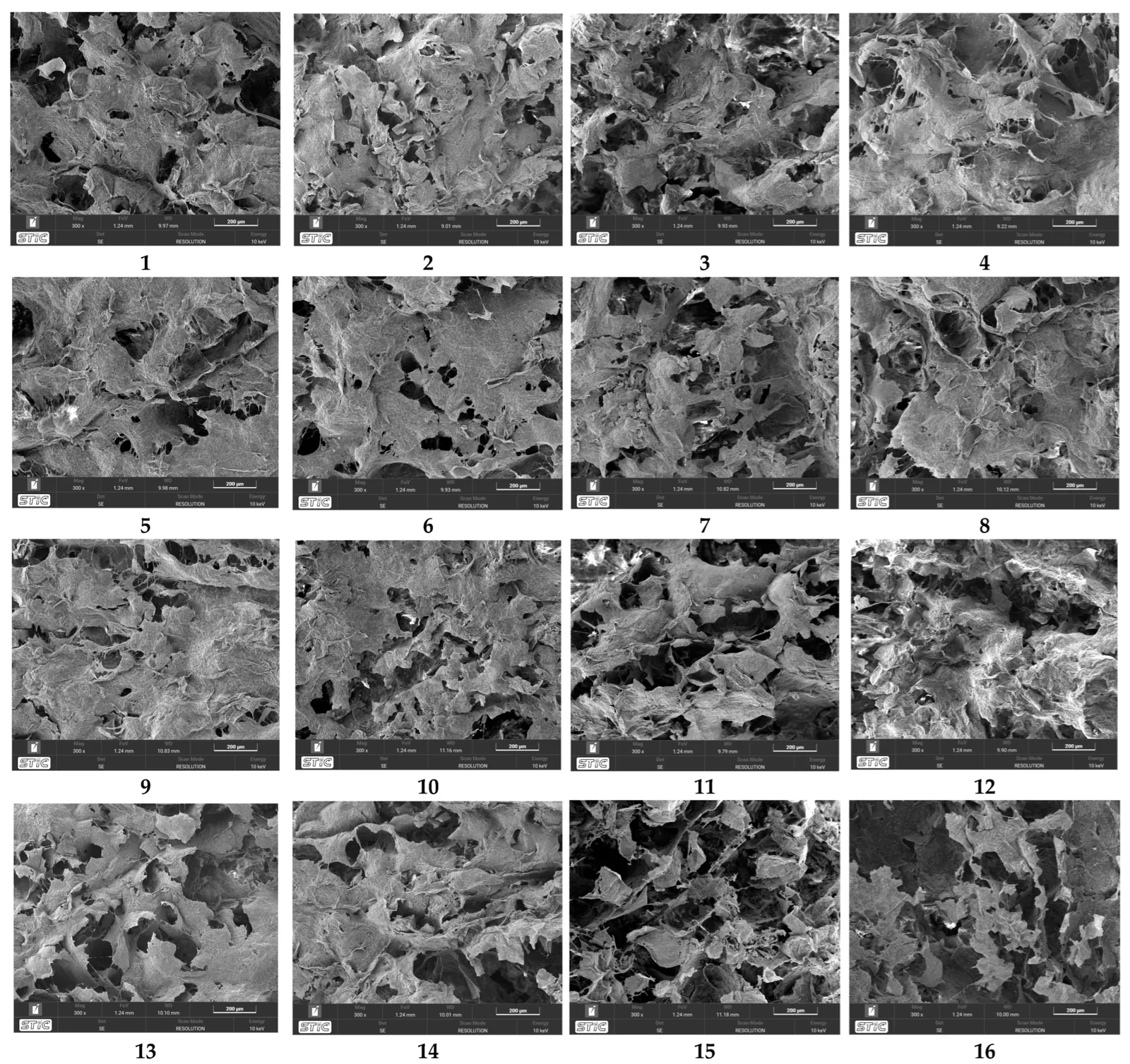
SEM micrographs of the top surface of 3D-printed Kai-based formulations (Runs 1–16). magnification: 300×.

**Figure 15:**
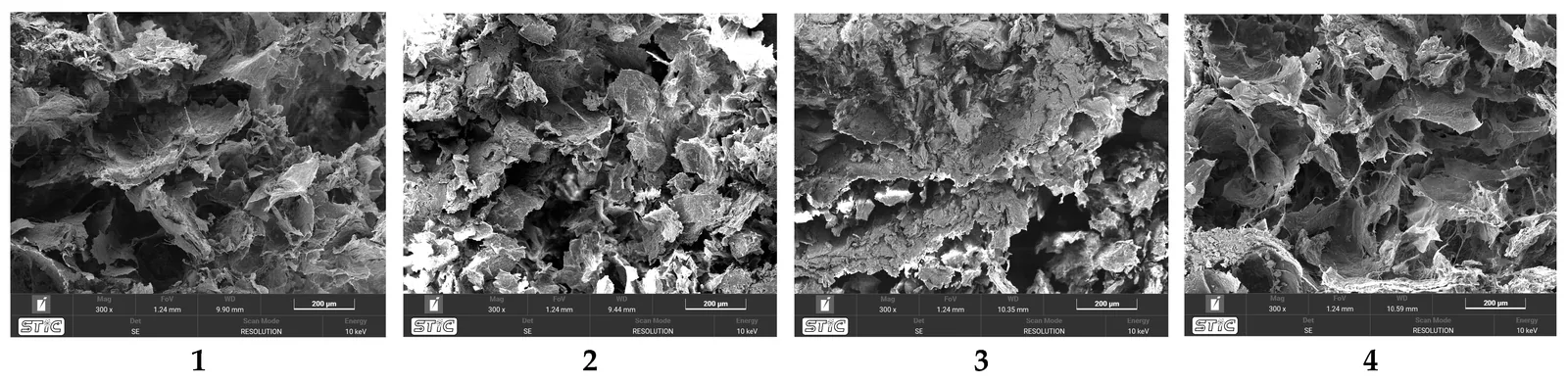

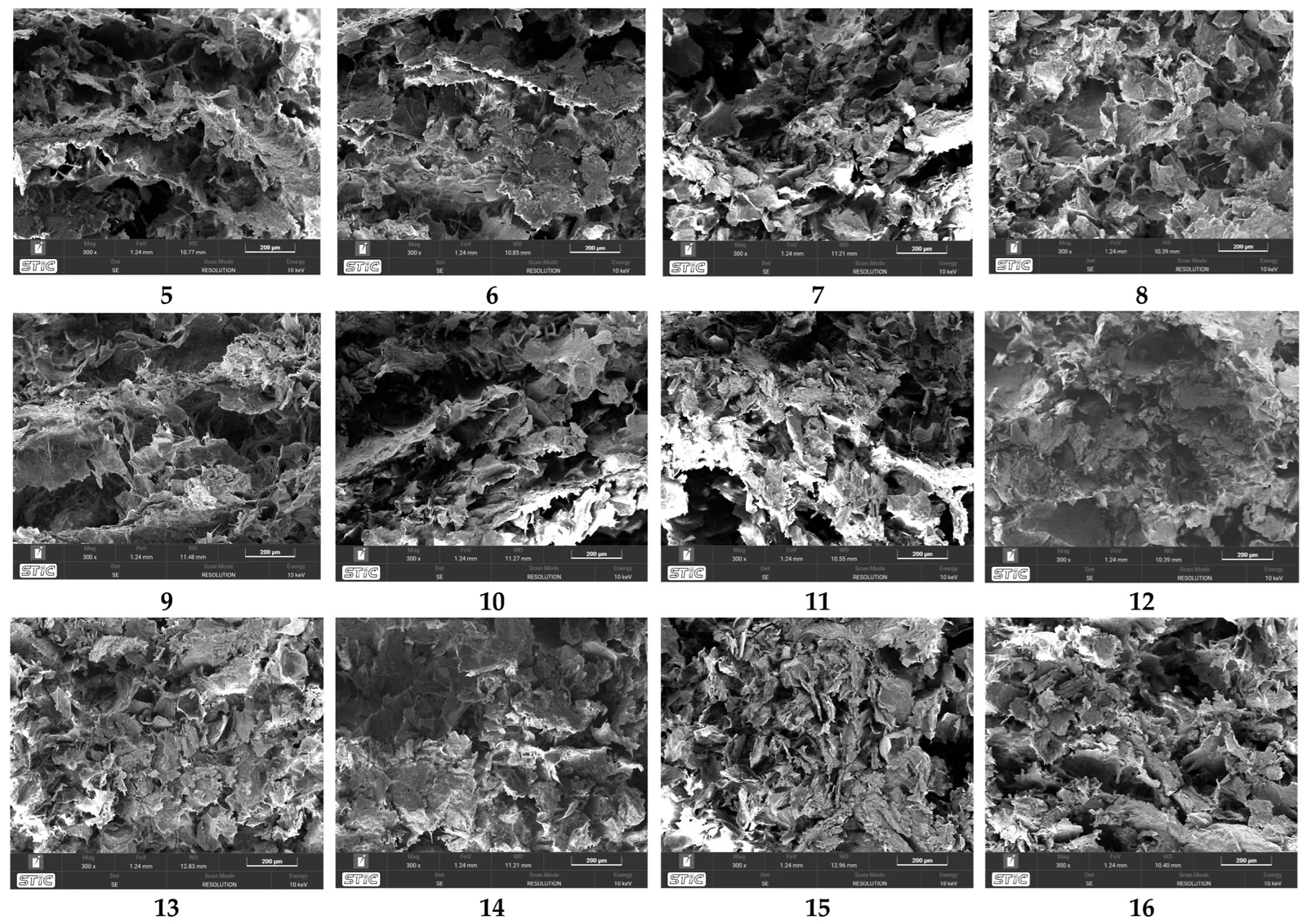
SEM micrographs of the cross section of 3D-printed Kai-based formulations (Runs 1–16). magnification: 300×.

The microstructure of the 16 printed formulations was examined by SEM at 300× magnification for the top surface (Figure 14) and the cross-section (Figure 15). Both views were analyzed because surface topography reflects extrusion quality and printing fidelity, while the cross-section reveals the internal polymer network that governs mechanical strength, water immobilization and shape stability [21,44].

The samples which contain only XG (Runs 13, 5, 6, 9) showed a progressive trend: as XG concentration increased, the top surface became smoother, and the cross-section evolved from a dense matrix to one containing visible pores. XG alone generally forms weak structured networks rather than strong thermally induced gels; however, its ordered helical conformation can contribute to network formation when combined with mannose-based polysaccharides such as KG [65,66]. In the absence of KG, however, pore structures remained incomplete. The KG-only sample (Run 2) had a smooth but fibrous surface and a dense, non-porous cross-section. Under the present formulation conditions, KG alone may exhibit limited gel network formation because native acetyl groups hinder intermolecular association and junction zone development [45,67].

Low-gum formulations (Runs 3 and 12) exhibited rough surfaces and dense cross-sections with visible gaps, indicating that insufficient polymer concentration cannot sustain a continuous network [16,55]. KG-dominant systems (Runs 7, 10 and 16) displayed surfaces ranging from medium-smooth to very rough, and cross-sections with incomplete pore development, large irregular voids or phase separation. KG-rich blends may promote localized polymer-rich regions associated with their water retention properties, which can contribute to increased adhesiveness and heterogeneous structures [21]. The XG-dominant formulation (Run 15) had a rough surface with holes but a dense cross-section; without sufficient KG to bridge the XG helices, an open pore architecture does not develop, and print accuracy remains limited.

Formulations with balanced XG:KG ratios produced clearly different microstructures. Runs 1 and 8 (≈1:2) showed relatively smooth surfaces with holes and relatively open pores structure with less defined pore walls in cross-section. Runs 11 and 14 (≈1:1) displayed small, uniform surface pores and a porous architecture with well-defined pore boundaries. Run 4 (≈3:1) had a smooth surface with uniform pores and more pronounced pore walls. These differences in morphology may be associated with interactions between XG and KG, as appropriate combinations of these hydrocolloids have been reported to enhance gel strength and network formation [18,21,68].

Only formulations with total gum ≥ 6% and XG:KG between 1:2 and 3:1 developed a uniform, well interconnected porous network. These microstructures corresponded to the highest print accuracy, lowest deformation, low syneresis and successful IDDSI testing. Formulations outside this compositional window showed dense, collapsed or phase-separated structures and showed less favorable performance in IDDSI-based texture assessment. The results confirm that microstructure is the physical link between hydrocolloid composition and functional performance in 3D-printed dysphagia foods.

## 4. Conclusions

This study demonstrated that XG–KG composition strongly influenced the textural, rheological, microstructural, and printing properties of *C. glomerata*-based formulations. XG primarily contributed to hardness, whereas KG enhanced cohesiveness and adhesiveness, with balanced formulations exhibiting favorable shear-thinning behavior, low syneresis, improved print accuracy, and reduced deformation. Several formulations met established criteria for dysphagia-modified foods, and numerical optimization identified a formulation containing 5.263% XG and 3.188% KG with high predicted print accuracy (95.18%) and desirability (0.91). These findings support the potential of *C. glomerata* as a sustainable ingredient for 3D-printed dysphagia foods and provide a basis for further experimental validation. Although the formulations were assessed against established dysphagia-related texture criteria, sensory acceptability, long-term storage stability, and clinical swallowing performance were not evaluated in this study and require further investigation before practical application.

## Figures and Tables

**Table 1:** Experimental conditions (RSM).

Run	Factor 1 (A: XG %)	Factor 2 (B: KG %)
1	2.070	4.320
2	0	9
3	0.609	2.391
4	6.910	2.090
5	5.265	0
6	5.265	0
7	0.225	5.67
8	2.070	4.320
9	9	0
10	0.225	5.670
11	4.365	4.148
12	0.609	2.390
13	3	0
14	4.365	4.148
15	4.14	1.935
16	2.430	6.570

**Table 2:** Optimization criteria and constraints applied in RSM desirability analysis.

Variable	Type	Goal	Range
XG (%)	Factor	In range	0–9
KG (%)	Factor	In range	0–9
Hardness (N/m^2^)	Response	Target range	2500–10,000
Adhesiveness (J/m^3^)	Response	Target range	0–400
Cohesiveness	Response	Target range	0.2–0.6
Print Accuracy (%)	Response	Maximize	91.87–96.10
Syneresis (cm^2^)	Response	Minimize	1.54–2.84
Deformation (%)	Response	Minimize	0.07–2.39

The target ranges for hardness, adhesiveness, and cohesiveness were selected according to Regulation I (serious swallowing difficulty) of the Japanese dysphagia food criteria [11].

**Table 3:** Experimental design and measured responses of Kai inks.

Factor		Response					
XG (%)	KG (%)	Hardness (N/m^2^)	Adhesiveness (J/m^3^)	Cohesiveness	Print Accuracy (%)	Syneresis (cm^2^)	Deformation (%)
2.070	4.320	1082.01 ± 40.00	168.01± 14.71	0.5753 ± 0.1789	94.07	2.27	0.92
0	9	604.23 ± 20.02	188.33 ± 12.63	0.5699 ± 0.0997	94.08	2.27	1.89
0.609	2.391	954.46 ± 14.46	153.14 ± 7.28	0.4122 ± 0.0397	92.66	2.72	2.39
6.910	2.090	3310.52 ± 9.66	187.01 ± 3.33	0.4894 ± 0.0900	94.65	2.27	0.49
5.265	0	986.15 ± 14.61	165.06 ± 9.76	0.4469 ± 0.0191	93.02	2.27	1.64
5.265	0	954.96 ± 25.38	165.25 ± 8.10	0.4458 ± 0.0109	93.16	2.27	1.06
0.225	5.67	1050.78 ± 39.26	175.07 ± 8.47	0.6281 ± 0.0299	94.19	2.84	1.22
2.070	4.320	1079.18 ± 41.36	172.17 ± 14.03	0.5781 ± 0.0259	94.19	2.27	0.83
9	0	1050.45 ± 22.30	162.44 ± 8.02	0.4894 ± 0.0210	94.45	1.77	0.22
0.225	5.670	1114.12 ± 37.30	172.55 ± 8.91	0.6256 ± 0.0133	94.25	2.55	1.31
4.365	4.148	2132.74 ± 8.54	183.09 ± 5.15	0.5742 ± 0.0019	96.10	1.77	0.07
0.609	2.390	954.16 ± 14.88	140.07 ± 10.02	0.4998 ± 0.0044	93.02	2.72	2.03
3	0	954.96 ± 22.76	126.45 ± 2.08	0.4263 ± 0.0099	91.87	2.27	2.19
4.365	4.148	2355.56 ± 18.95	180.99 ± 4.05	0.5788 ± 0.0010	96.01	1.54	0.19
4.14	1.935	1909.92 ± 6.90	165.20± 15.60	0.4933 ± 0.0063	93.15	2.27	0.94
2.430	6.570	1273.28 ± 44.95	189.13± 12.25	0.6647 ± 0.0200	94.50	2.27	0.39

Values are presented as mean ± SD (*n* = 3) for directly measured responses. For print accuracy, deformation, and syneresis, the corresponding dimensions were measured in triplicate, and the mean values were used in the respective equations to calculate the final response values.

**Table 4:** Summary statistics of measured responses.

Response	Min	Max	Mean	Standard Deviation
Hardness (N/m^2^)	604.2	3310	1360	706.1
Adhesiveness (J/m^3^)	126.4	189.1	168.4	17.29
Cohesiveness	0.4122	0.6647	0.5311	0.0780
Print Accuracy (%)	91.87	96.10	93.96	1.13
Syneresis (cm^2^)	1.539	2.835	2.27	0.3497
Deformation (%)	0.071	2.395	1.15	0.76

**Table 5:** Regression coefficients and model statistics for models.

	Hardness	Adhesiveness	Cohesiveness	Print Accuracy	Syneresis	Deformation
Model	Reduced Cubic	Reduced Cubic	Reduced Cubic	Reduced Quadratic	Cubic	Reduced Quadratic
Intercept	582.1	126.3	0.4062	90.68	−15.35	3.406
*x*_1_	133.3 ***	−5.393 ***	0.008279 *	0.4128 ***	11.13 **	−0.3379 ***
*x*_2_	237.9 **	10.32 ***	−0.01566 ***	0.9251 ***	10.98 **	−0.6094 ***
*x*_1_ *x*_2_	−86.84 ***				−3.941 **	
*x*_1_^2^	−8.971 ***	3.821			−2.118 ***	
*x*_2_^2^	−25.53 *	−0.3488	0.01909 ***	−0.06136 **	−1.993 **	0.0465 ***
*x*_1_^2^ *x*_2_	30.36 ***				0.3379 ***	
*x*_1_ *x*_2_^2^					0.3225 ***	
*x*_1_^3^		−0.3084 *			0.1214 **	
*x*_2_^3^			−0.001701 ***		0.1101 ***	
*R* ^2^	0.9750	0.9144	0.9472	0.8456	0.9628	0.9314
Adjusted *R*^2^	0.9583	0.8716	0.9279	0.8070	0.9070	0.9143
Predicted *R*^2^	0.9073	0.7185	0.8712	0.7679	0.8443	0.7366
Adequate Precision	27.09	14.49	19.95	13.49	12.63	21.58
Model df	6	5	4	3	9	3
Model *F*-value	58.51	21.37	49.29	21.91	17.26	54.34
Model *p*-value	<0.0001	<0.0001	<0.0001	<0.0001	0.0012	<0.0001
Residual df	9	10	11	12	6	12
Pure error df	5	5	5	5	5	5
Lack of Fit df	4	5	6	7	1	7
Lack of Fit *F*-value	7.30	2.86	0.2102	23.72	0.0041	4.51
Lack of Fit *p*-value	0.0256	0.1370	0.9580	0.0015	0.9516	0.0582

Regression coefficients are presented in coded units. * *p* < 0.05; ** *p* < 0.01; *** *p* < 0.001; terms with *p* ≥ 0.05 were considered not significant. *R*^2^, coefficient of determination; Adeq. Precision, Adequate Precision. Model and lack-of-fit statistics were obtained from analysis of variance (ANOVA).

**Table 6:** Cross-framework classification matrix combining Japanese standard regulation with UDF and IDDSI Level 4 requirement.

Formulation Group	Japanese Regulation	UDF	Observation Fork and Spoon Tests and Printed Sample	IDDSI Level 4 Status	Suitable for Application
Run 4	I	4	Fork: The sample remained cohesive on the fork without flowing through the tines. Spoon: The sample maintained its shape and slid off the spoon cleanly with minimal residue.	PASS	YES
Runs 1, 8, 10, 11, and 14	II	4	Fork: Samples retained their structure on the fork without dripping through the slots. Spoon: Samples formed a cohesive bolus and detached cleanly from the spoon surface.	PASS	YES
Run 15	II	4	Fork: The sample remained stable on the fork without dripping. Spoon: The sample detached cleanly from the spoon surface Note: Not smooth surface and poor 3D-printing accuracy (93.15%) limited its suitability for final application.	PASS	NO (Printing limitation)
Runs 7, 9, and 16	II	4	Fork: Samples remained stable on the fork without dripping through the tines. Spoon: Samples failed the spoon tilt test due to excessive elasticity, resulting in stretching and strong adhesion to the spoon surface.	FAIL	NO
Runs 2, 3, 5, 6, 12, and 13	III	4	Fork: Samples initially retained their mass on the fork. Spoon: Samples failed the spoon tilt test because the weak structure collapsed and spread across the spoon surface instead of remaining cohesive.	FAIL	NO

## Data Availability

The original contributions presented in the study are included in the article. Further inquiries can be directed to the corresponding author.

## Abbreviations

The following abbreviations are used in this manuscript:

3D	Three dimensional
CAA	Consumer Affairs Agency (Japan)
IDDSI	International Dysphagia Diet Standardization Initiative
KG	Konjac gum
MHLW	Ministry of Health, Labor and Welfare (Japan)
RSM	Response surface Methodology
SEM	Scanning electron microscopy
TPA	Texture profile analysis
UDF	Universal Design Food
XG	Xanthan gum
